# Integrative Genomics-Based Discovery of Novel Regulators of the Innate Antiviral Response

**DOI:** 10.1371/journal.pcbi.1004553

**Published:** 2015-10-20

**Authors:** Robin van der Lee, Qian Feng, Martijn A. Langereis, Rob ter Horst, Radek Szklarczyk, Mihai G. Netea, Arno C. Andeweg, Frank J. M. van Kuppeveld, Martijn A. Huynen

**Affiliations:** 1 Centre for Molecular and Biomolecular Informatics, Radboud Institute for Molecular Life Sciences, Radboud university medical center, Nijmegen, The Netherlands; 2 Virology Division, Department of Infectious Diseases and Immunology, Faculty of Veterinary Medicine, University of Utrecht, Utrecht, The Netherlands; 3 Department of Internal Medicine and Radboud Center for Infectious Diseases, Radboud university medical center, Nijmegen, The Netherlands; 4 Department of Viroscience, Erasmus Medical Center, Rotterdam, The Netherlands; La Jolla Institute for Allergy and Immunology, UNITED STATES

## Abstract

The RIG-I-like receptor (RLR) pathway is essential for detecting cytosolic viral RNA to trigger the production of type I interferons (IFNα/β) that initiate an innate antiviral response. Through systematic assessment of a wide variety of genomics data, we discovered 10 molecular signatures of known RLR pathway components that collectively predict novel members. We demonstrate that RLR pathway genes, among others, tend to evolve rapidly, interact with viral proteins, contain a limited set of protein domains, are regulated by specific transcription factors, and form a tightly connected interaction network. Using a Bayesian approach to integrate these signatures, we propose likely novel RLR regulators. RNAi knockdown experiments revealed a high prediction accuracy, identifying 94 genes among 187 candidates tested (~50%) that affected viral RNA-induced production of IFNβ. The discovered antiviral regulators may participate in a wide range of processes that highlight the complexity of antiviral defense (e.g. *MAP3K11*, *CDK11B*, *PSMA3*, *TRIM14*, *HSPA9B*, *CDC37*, *NUP98*, *G3BP1*), and include uncharacterized factors (*DDX17*, *C6orf58*, *C16orf57*, *PKN2*, *SNW1*). Our validated RLR pathway list (http://rlr.cmbi.umcn.nl/), obtained using a combination of integrative genomics and experiments, is a new resource for innate antiviral immunity research.

## Author Summary

Viruses pose a continuous threat to human health, even though our immune systems have evolved to neutralize invading viruses. As part of the innate immune system, the RIG-I-like receptors (RLRs) are essential for detecting viruses during infection. Recognition of viral RNA by the RLRs triggers an antiviral response that inhibits viral replication, protects uninfected cells, and attracts specialized immune cells. Better understanding of the innate antiviral response may reveal novel targets for antiviral therapeutics and vaccine development. However, that requires knowledge about which genes and proteins are involved. In the present study, we systematically investigated the wealth of available genomics data (including gene expression, protein interactions, transcription regulation and genome sequences) and discovered no less than 10 distinctive properties of genes known to be part of the antiviral RLR pathway. By combining these properties in a statistical framework, we predicted 187 novel RLR pathway components. Our validation experiments showed that ~50% of the predicted candidate genes have a significant effect on antiviral signaling. These results, together with independent computational and literature-based confirmation, demonstrated the validity of our combined bioinformatics and experimental approach. Our study expands the collection of known antiviral genes, opening up new avenues for research into innate antiviral immunity.

## Introduction

Viruses are a major cause of human disease, as highlighted by the pandemics of influenza viruses, HIV–1, and the current outbreak of the Ebola virus. Pattern recognition receptors (PRR) are among the first molecules that detect viruses during infection. The RIG-I-like receptors (RLRs, one class of PRRs) are part of the RLR pathway, which forms a crucial innate antiviral defense system [1,2]. Two RLRs, RIG-I and MDA5, reside in the cytosol where they recognize non-self 5’-triphosphate RNA molecules with short double-stranded regions and long double-stranded RNAs (dsRNA), respectively [3]. Activation of the receptors triggers a complex signaling network, key steps of which are the activation of the mitochondrial adapter MAVS, subsequent recruitment of the TBK1 and IKK complexes, phosphorylation/activation of IRF3 and NFκB, and translocation of these transcription factors to the nucleus. These steps ultimately lead to the production of type I interferons (IFNα/β) and proinflammatory cytokines, which are crucial for establishing an antiviral state in infected as well as neighboring cells, and also modulate the adaptive immune response [4]

The importance of the RLR system is further demonstrated by the observation that viruses of all types employ strategies to interfere with its activation, often at multiple steps [5,6]. Better understanding of viral interaction with the pathway has resulted in novel targets for the development of antiviral therapeutics and attenuated live vaccines, for example viruses lacking functional RLR antagonists [7]. Furthermore, mutations in RIG-I, MDA5, MAVS and other RLR pathway components are associated not only with strong susceptibility to infections, but also IFN-associated autoimmune disorders [8–10].

Previous studies into virus-host interactions and the innate antiviral pathways have used genomics approaches, often generating large data sets describing physical or genetic interactions [11–14]. Other publications have taken a comparative approach based on model organisms [15] or used over-expression screening systems [16,17]. Together, these studies have identified numerous genes with antiviral activity, including members of the RLR pathway. However, it remains important to systematically assess the quality of individual data sets as such screens report distinct sets of genes, often with limited overlap between them. Combining the many available genomics data sets in a statistical framework potentially allows for a more systematic discovery and categorization of genes involved in the RLR pathway. Indeed, Bayesian integration of large-scale data that includes weighing individual datasets for their predictive potential has been successful in other cellular systems, for example identifying novel protein interactions [18], mitochondrial disease genes [19], and small RNA pathway genes [20].

In this work we systematically exploit the wealth of available (gen)omics data, including transcriptomics and proteomics data, genome sequences, protein domain information, and functional genomics, to discover descriptive molecular signatures of the RLR pathway system. Bayesian integration of these data, together with comprehensive computational and experimental validation, confidently identifies novel genes involved in antiviral RIG-I signaling.

## Results

The RIG-I-like receptor (RLR) pathway is a highly interconnected and diverse molecular system. We investigated whether available genomics data contain sufficient signal to accurately describe RLR pathway components, and whether such data could be used to prioritize novel genes for a possible role in RLR signaling.

### Ten molecular signatures of RLR pathway components in genomics data

To discover molecular signatures that distinguish RLR pathway components from other genes, we explored a wide variety of genome-scale data describing different aspects of the virology and biology of the pathway. Some of these data we used directly, while other data were used as the basis for further calculations (**Table 1**). We quantitatively assessed the predictive power of each data set using a literature-curated standard of 49 known RLR pathway components from InnateDB [21] (‘RLR genes’, **S1 Fig**) and a set of 5,818 ‘non-RLR genes’ that are unlikely to be part of the pathway (i.e. genes with known functions not directly related to the innate antiviral response, such as development, housekeeping and neurological processes, see **Methods**). Below we describe 10 signatures for predicting novel RLR pathway components. The first five signatures are based on the relationship of RLR genes with viruses, whereas the second set of five signatures are based on properties of the RLR pathway itself.

**Table 1:** Ten molecular signatures from genomics data used for predicting novel RLR pathway components.

Group	Molecular signature	Data set description	Type ^a^	References	Number of genes ^b^	Likelihood ratio score ^b^ ^,^ ^c^
Virus-based	Positive selection in primates	Rapidly evolving genes in the primates lineage, detected using maximum likelihood analysis of nucleotide alignments	d	[23]	926	1.7
	PPI with viruses	Virus-interacting human proteins extracted from PPI databases	**c**	[28]	2,587	4.2
	Viral miRNA target	Likelihood scores of targeting of human transcripts by viral miRNAs, based on predicted target sites	**c**	[30]	6,761	1.3
	Differential expression upon infection	Genes showing differential expression in lung epithelial cells infected with four respiratory viruses	**c**	see **Methods**	1,680	3.5
	Antiviral host factor	Meta analysis of genes with antiviral activity from seven RNAi screens studying a variety of viruses	**c**	see **S3 Table**	173	2.1
Pathway-based	Co-expression with RLR pathway	Weighted co-expression with known RLR genes across >450 human gene expression studies	**c**	[32]	4,149	2.4
	RLR pathway protein domain	Proteins containing one of the 25 domains that are significantly enriched in known RLR proteins	**c**	[66]	711	8.9
	Innate antiviral TF binding motifs	Genes with IRF, AP–1, NFκB, or STAT TF binding motifs in their promoters, based on conservation across 29 mammals	**c**	[33]	4,508	2.3
	NFκB activation mediator	Hits from a genome-wide siRNA screen of Epstein-Barr virus-induced NFκB activation	d	[34]	154	19.8
	RLR pathway PPI	Proteins that interact with known RLR proteins, calculated from PPI data	**c**	[35]	1,750	4.3
**Integration**	**RLR score**	**Bayesian integration of the 10 molecular signatures to predict novel RLR pathway components**				

^a^ Data used directly (d) or as basis for further calculation (c)

^b^ Combination of all bins with positive likelihood ratio scores per feature, derived from **Fig 1A**

^c^ RLR genes versus non-RLR genes: *P*(D_i_ | RLR genes) / *P*(D_i_ | non-RLR genes), see **Methods**.

Note that, to avoid circularity, the predictive ability of the co-expression, protein domain and RLR pathway PPI data sets was assessed using the set of TLR, CLR, NLR, cytDNA genes instead of the RLR genes (see **Methods**).

**Fig 1:**
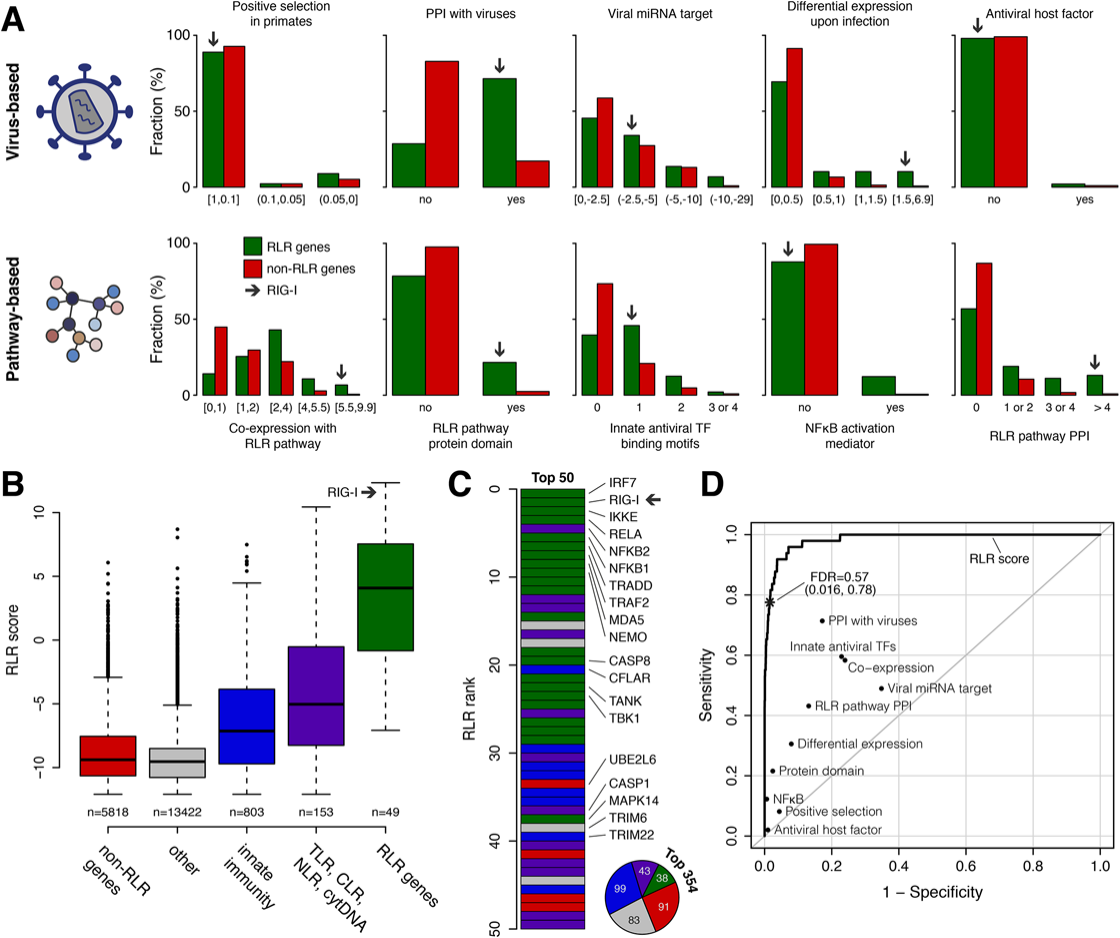
Bayesian integration of ten molecular signatures of RLR pathway components from genomics data. (**A**) Distributions of the 49 known RLR pathway components (RLR genes, green) and 5,818 genes unlikely to be part of the pathway (non-RLR genes, red) across the 10 molecular signature data sets we identified as predictive of the RLR system (see also **Table 1**). Data sets were binned into discrete intervals and fractions of (non-)RLR genes add up to one. Arrows indicate the behavior of RIG-I across the data. The top five signatures describe the relationship of RLR signaling with viruses; the bottom five describe properties of the pathway itself. (**B**) Boxplots of the genome-wide integrated RLR score (Bayesian posterior probability score). Genes were grouped into one of five classes: known RLR genes (green, see [A]), components of other PRR signaling pathways (‘TLR, CLR, NLR, cytDNA’; purple), genes functioning in other aspects of the innate immune response (‘innate immunity’; blue), and non-RLR genes (red, see [A]). The remaining genes are classified as ‘other’ (gray). (**C**) The 50 genes with the highest RLR scores. Representative RLR and other innate antiviral response genes are indicated. The pie chart shows the occurrences of the different gene classes in the top 354 RLR ranks. (**D**) Receiver operating characteristic (ROC) curve illustrating the performance of the integrated RLR score (solid black line) and the individual molecular signatures (black dots) for predicting known RLR versus non-RLR genes. Sensitivity and specificity were calculated at various score thresholds (for the RLR score), or at specific thresholds that include all bins with positive likelihood ratio scores (for the individual data sets; see (A)). The asterisk denotes the sensitivity and specificity corresponding to a false discovery rate (FDR) of 57% (top 354 genes). Note that, to avoid circularity, the predictive ability of the co-expression, protein domain and RLR pathway PPI data sets in (A) and (D) was assessed using the set of TLR, CLR, NLR, cytDNA genes instead of the RLR genes (see **Methods**).

See also **Fig 1A** and **S1 Table**.

### Virus-based signatures

#### Positive selection in primates

Viruses evade recognition or interfere with the immune systems of their hosts to achieve successful infection [7]. This involves interactions between viral and host proteins, the interfaces of which are under constant pressure to change [22]. We collected data on recurrent positive selection in the primate lineage, based on maximum likelihood analysis of sequence alignments [23]. RLR pathway components, e.g. the mitochondrial signaling adapter MAVS [24] and transcription factor IRF7 [25], are enriched for rapidly evolving genes (9% of 49 RLR genes) compared to genes that are unlikely to be part of the pathway (5% of 5,818 non-RLR genes, 1.7-fold enrichment, non-significant *P* = 0.38, one-tailed Fisher’s exact test, **Fig 1A**, **Tables 1**and **S1**).

#### Protein-protein interactions (PPI) with viruses

The next signature is based on the physical interactions between host RLR pathway components and viruses. Viral proteins often interact with many host proteins during their infection cycle, including those involved in antiviral defense [26,27]. Extraction of virus-human PPIs from specialized databases [28] revealed ~2,600 human proteins that are reported to interact with at least one viral protein (virus-interacting human proteins, **S2 Fig**). These virus-interacting human proteins include the majority of RLR pathway components (35/49 = 71%), while they include a significantly smaller fraction of non-RLR genes (1,000/5,818 = 17%, 4.2-fold enrichment, *P* = 1.4 × 10^−16^, one-tailed Fisher’s exact test, **Fig 1A** and **Table 1**). Among the RLR genes, TRAF2 (4 PPIs), DDX3, MAPK9, and the NFκB subunit RELA (3 PPIs each) have reported interactions with the largest number of distinct virus species.

#### Viral miRNA target

Another mechanism that viruses use to interfere with the antiviral activity of human cells is down-regulation of gene expression by miRNAs [29]. We collected 128 miRNAs encoded by nine, mainly herpes DNA viruses (**S2 Table**) [30], most of which have confirmed physiological relevance. Predicted target sites of these miRNAs to the 3’UTR of human transcripts were then used to calculate for each gene a score representing the likelihood that viruses affect its expression (viral miRNA targeting score). For example, our method assigned *IKBKE* (IKKε) a relatively strong viral miRNA targeting score of 2.7. Indeed, Kaposi’s sarcoma-associated herpesvirus miR-K12-11 has been shown to inhibit translation of IKKε transcripts, leading to suppression of interferon signaling [31]. Analysis of the viral miRNA targeting scores revealed that RLR genes tend to have stronger scores than non-RLR genes (*P* = 0.03, one-tailed Mann-Whitney *U* test). Although the statistical significance of this trend is only marginal, we included it as a molecular signature of RLR genes as it still provides a moderate enrichment over non-RLR genes (1.3-fold enrichment among genes with the strongest viral miRNA targeting scores, **Table 1**), and even weak features can substantially improve the predictions for novel RLR genes.

#### Differential expression upon infection

Next, we asked whether RLR pathway genes are differentially expressed upon virus infection. To answer this, we used in-house gene expression data of human lung epithelial cells (A549) exposed to four respiratory viruses (respiratory syncytial virus, human metapneumovirus, parainfluenza virus, or measles virus), for which gene expression was measured at 6, 12 and 24 hours after infection (see **Methods**). Analysis of the transcriptomes revealed that many RLR genes (31%) underwent substantial expression changes (log_2_ fold change >0.5) in cells infected with the respiratory viruses, compared to the uninfected cells. This compares to a much smaller fraction of non-RLR genes (9%, 3.5-fold enrichment, *P* = 1.3 × 10^−5^, one-tailed Fisher’s exact test, **Fig 1A** and **Table 1**). The differentially expressed RLR genes include well-known interferon-stimulated genes (ISGs) like *ISG15*, *DDX58* (RIG-I), *IRF7*, *IFIH1* (MDA5), and *TRIM25*, which are the top five RLR genes most induced by the respiratory viruses studied (log_2_ fold change >1.5 compared to uninfected cells, **S3 Fig**). Thus, even though we expect many RLR genes to already be expressed before viral infection, their expression levels are reinforced in infected cells.

#### Antiviral host factor

RNAi screening potentially allows the identification of host factors that *limit* virus replication, such as genes involved in the innate antiviral response, although most studies focus on hits with the opposite effect (i.e. factors *required* by viruses for replication) [11]. We performed a meta analysis of hits from seven large-scale RNAi studies in human cells, identifying 173 genes with antiviral activity against HIV–1, influenza, hepatitis C (HCV), West Nile, or enterovirus infection (**S3 Table**). In contrast to our expectation that RLR genes would be common among these antiviral host factors, this data set is one of the weaker predictors of RLR genes: the 173 antiviral host factors contain only a single gene (IRF3) that belongs to the set of 49 known RLR genes (~2%) compared to 56 of 5,818 non-RLR genes (~1%, 2.1-fold enrichment, non-significant *P* = 0.38, one-tailed Fisher’s exact test, **Fig 1A** and **Table 1**).

### Pathway-based signatures

#### Co-expression with RLR pathway

To aid in finding novel RLR genes, we screened >450 human expression studies for genes that co-express with known RLR pathway components using a two-step approach [32]. First, we weighed individual expression data sets for their propensity to predict new RLR genes: experiments in which the whole group of known RLR genes show high co-expression with each other received a higher weight and contributed most to the calculations. In the second step, we calculated the co-expression of all genes with the RLR genes. As expected, RLR genes display significantly higher co-expression scores with each other than with the rest of the genome or with non-RLR genes (*P* ≈ 10^−27^ for both comparisons, one-tailed Mann-Whitney *U* test, **S4 Fig**). However, RLR genes also score higher than components of other PRR signaling pathways (Toll-like receptor [TLR], C-type lectin receptor [CLR], NOD-like receptor [NLR], and cytosolic dsDNA sensing [cytDNA] pathways; *P* = 4.5 × 10^−13^, one-tailed Mann-Whitney *U* test). Cross-validation by leave-one-out analysis confirms that the weighted co-expression approach retrieves RLR genes more readily than other PRR pathway genes, or genes involved in other aspects of innate immunity (**S4D Fig**), demonstrating specificity for identifying RLR genes in the co-expression data.

#### RLR pathway protein domain

Analysis of RLR pathway protein sequences revealed the presence of 40 unique domains, 25 of which were significantly over-represented compared to the full human proteome (Benjamini-Hochberg-corrected Fisher’s exact *P* < 0.01, **S4 Table**). These include protein kinase domains (12-fold enrichment, *P* = 1.1 × 10^−8^; present in IKKα/β/ε, MAP kinases, TBK1), the TBK1/IKKi binding domain (TANK, TBK1BP1, AZI2), caspase and death domains (CASP8/10, FADD), IRF domains (IRF3/7), and the DExD/H box RNA helicase domain (15-fold enrichment, *P* = 2.7 × 10^−3^; RIG-I, MDA5, LGP2). We then assessed the domain organizations of all human proteins and determined a set of 711 proteins containing one or more of the domains enriched in RLR components. These proteins are predictive for RLR components with an enrichment score of 8.9 (**Table 1**).

#### Innate antiviral transcription factor (TF) binding motifs

Signaling through the RLR pathway triggers the activation of key transcription factors (TFs) like IRF3, IRF7, AP–1 and NFκB. Activation of these TFs leads to the production of type I interferons and proinflammatory cytokines that eventually activate STAT1 and STAT2 [2]. STAT1 and STAT2 in turn stimulate transcription of interferon-stimulated genes (ISGs), which include many RLR pathway components. To further explore the transcription regulation of RLR pathway components, we analyzed their gene promoters for the presence of TF binding motifs that are highly conserved across the genomes of 29 placental mammals, such as primates, rodents and many farm animals [33]. Conserved IRF and NFκB motifs are highly abundant in the promoters of RLR genes (Fisher’s exact *P* = 3.3 × 10^−3^ and *P* = 2.0 × 10^−4^, respectively; **S5 Table**), suggesting the pathway is partly self-regulating as has been observed for individual components. Interestingly, a conserved IRF motif was detected not only in the promoters of *IRF7* itself and in all three RIG-I-like receptor family members (*DDX58* [RIG-I], *IFIH1* [MDA5], *DHX58* [LGP2]), but also in *TRIM25*, *ISG15*, and *CYLD*; three factors controlling RIG-I signaling activation by regulating the level of K63 polyubiquitination. In order to predict novel RLR components, we searched for genes containing conserved IRF binding motifs (several motif variants, collectively recognized by IRF1-9), STAT binding motifs (several motif variants, collectively recognized by STAT1-6), AP–1 binding motifs, or NFκB binding motifs (**Fig 1A**). We found 3,558 genes across the human genome containing one of these motifs in their promoters. This large number partially stems from similarities in the DNA binding preferences of TFs that belong to the same family, but does not mean that all identified genes are regulated by the RLR pathway. For example, STAT motifs not only occur in the promoters of ISGs, but also in genes involved in cellular proliferation, differentiation and apoptosis. Nevertheless, genes containing one of the four conserved TF motifs already show a good predictive value for RLR pathway components (enrichment score of 2.2, **S1 Table**). Genes with more than one motif are even more likely to be RLR genes: 789 genes contain two motifs (2.6-fold enrichment) and 161 genes contain three or all four motifs (2.4-fold enrichment).

#### NFκB activation mediator

Host factors that regulate NFκB activation often also affect the RLR pathway. Indeed, the 154 hits that were picked up in a genome-wide siRNA screen of Epstein-Barr virus-induced NFκB activation [34] include a much larger fraction of known RLR genes (6/49 = 12%) than non-RLR genes (36/5,818 < 1%, 20-fold enrichment, *P* = 1.0 × 10^−6^, one-tailed Fisher’s exact test, **Fig 1A** and **Table 1**). Thus, these 154 NFκB activation mediators are likely to contain novel RLR pathway components as well.

#### RLR pathway PPI

Finally, to find novel RLR genes we assessed the human protein interaction network connecting the RLR pathway. PPI databases [35] report 3,504 interactions between 1,750 unique proteins and 47 of the 49 RLR components, the only exceptions being DAK and NLRX1. Of the 47 RLR proteins with reported PPIs, 41 are involved in a total of 147 interactions within the pathway (i.e. between two pathway members). This network of RLR components has significantly more connections with each other than do random networks of the same size and interaction degree distribution (physical interaction enrichment score = 3.6, *P* < 1.0 × 10^−6^ [36]). Using the PPI data, we obtained for each human protein the number of interacting RLR pathway components (**Fig 1A**). We found a total of 1,397 proteins reported to interact with one or two RLR proteins. These proteins are predictive for RLR components with an enrichment score of 1.8 (**S1 Table**). A further 221 proteins interact with three of four RLR proteins (6.3-fold enrichment) and 132 proteins interact with five or more RLR proteins (15.8-fold enrichment). TBK1 (18 interactions), TRAF2 and MAVS (both 16) top the list, supporting their roles as central players in the RLR system [12]. Thus, an increasing number of interactions with RLR proteins indicates a higher likelihood that a protein is part of the RLR pathway.

### Bayesian integration of molecular signatures provides genome-wide probabilities for RLR pathway components

The RLR pathway components published thus far probably constitute only part of the total proteins with a function in this pathway. To prioritize novel high-confidence genes for a role in the RLR pathway, we integrated the 10 identified molecular signatures of RLR genes in a naive Bayesian classifier [18,19] (see **Methods**). This approach weighs data sets based on their predictive value (i.e. their ability to separate known positives and negatives; **Fig 1A**, **Tables 1**and **S1**) so that ‘better’ data contribute more to the predictions. Each human gene received a posterior probability score (‘RLR score’) reflecting the likelihood that the gene is part of the RLR pathway based on its behavior in the collected genomics data. A score of zero indicates equal probabilities of a gene being an RLR versus a non-RLR gene. **S6 Table** presents the genome-wide ranking of RLR scores (also available at http://rlr.cmbi.umcn.nl/).

As expected, known RLR pathway components have the highest RLR scores (**Figs 1B** and **S5**). Two-thirds (32/49) of these rank within the first 150 genes. The top ranking genes are IRF7, RIG-I, IKKε, subunits of NFκB, TRADD, TRAF2, MDA5, and IKKγ (NEMO) (**Fig 1C**). Other examples of well-described RLR pathway components include IRF3 (rank 51), ISG15 (52), MAVS (102), and LGP2 (114). Genes that are unlikely to play a role in the pathway (the set of non-RLR genes) generally have very low RLR scores, although some of these received high scores as well (**Fig 1B**). This is not unexpected, as even though this large set of genes was selected from function annotations generally unrelated to the innate antiviral response, this does not preclude that individual genes (also) function in the RLR pathway.

To gain insight into what kind of genes are present among the RLR predictions, we examined their functions by pathway and gene ontology enrichment analyses. The top 354 genes with the highest RLR scores (corresponding to high-confidence predictions, see below) have strong links with other pathways of the innate immune response, such as TLR, NLR, interferon, and cytokine signaling (**S6**and **S7 Figs**). Antiviral defense functions are also among the most frequent and significant terms associated with the high-scoring genes (**S8 Fig** and **S7 Table**). Other important biological processes include various apoptosis-related functions, cancer and cell cycle pathways, and regulation of metabolic processes and protein localization. Furthermore, the top predictions include a wide range of protein families, notably proteasome subunits, ubiquitin(-like) conjugating enzymes, and genes involved in phosphatidylinositol signaling (which was recently shown to affect the type I IFN response [14]). Finally, 22% of the top predictions are induced in cells treated with interferons (i.e. they are interferon-stimulated genes, ISGs) and ~18% are part of the common host transcription response to pathogens (**Table 2**). Together, these observations indicate that our framework successfully predicts genes with a likely role in the innate antiviral response and suggests other cellular systems and functions required for this response.

**Table 2:** Overlap between innate (antiviral) response data sets and the top 354 RLR predictions excluding known RLR genes.

Data set	References	Number of genes in data set	Fraction (number) of data set genes in top 354 predictions	One-tailed Fisher’s exact *P*
Interferon-stimulated genes (ISGs)	[16]	354	22.0% (78)	1.2 × 10^−67^
ISGs with validated antiviral activity	[16,91]	45	42.2% (19)	4.9 × 10^−23^
Common host transcription response to pathogens	[92]	496	17.7% (88)	5.2 × 10^−68^
Interactors of the type I IFN protein network during pattern recognition (HCIP) ^a^	[12]	241	11.2% (27)	1.2 × 10^−15^
HCIP with confirmed effects on IFNβ expression and antiviral activity	[12]	22	22.7% (5)	1.9 × 10^−5^
Tripartite motif (TRIM) family genes	[17]	71	12.7% (9)	1.6 × 10^−6^
TRIMs that enhance RIG-I-induced activation of IFNβ, NFκB and ISRE promoters	[17]	34	14.7% (5)	1.7 × 10^−4^
Human interactors of innate immune-modulating viral ORFs ^b^	[37]	569	6.7% (38)	4.7 × 10^−14^
Genes expressed in PBMCs stimulated with *Candida* (CRG)	[38]	89	43.8% (39)	4.7 × 10^−47^
CRG with altered expression in CMC patients	[38]	23	65.2% (15)	2.6 × 10^−22^
Type I IFN response mediators	[14]	226	4.0% (9)	9.5 × 10^−3^

^a^ These PPIs were not part of the RLR interaction network used for the RLR predictions (i.e. for the ‘RLR pathway PPI’ signature)

^b^ These interactions were not used to determine the virus-interacting human proteins used for the RLR predictions (i.e. for the ‘PPI with viruses’ signature)

### Performance estimates and independent data establish the reliability of the RLR score

We further computationally assessed the reliability of the integrated RLR score by estimating the sensitivity, specificity and false discovery rate (FDR) of the predictions using the positive (RLR genes) and negative (non-RLR genes) standards. Integration of the data sets achieved better sensitivity and specificity than any of the individual data sets (**Fig 1D**), thereby enriching for RLR genes and depleting false positives (**S5**, **S9**and **S10 Figs**). At an RLR rank threshold of 354 (RLR score -1.10), the framework correctly predicts 78% of the known RLR genes with a specificity of 98.4% (**Fig 1D**). At this threshold, only ~57% of the novel predictions are estimated to be false (**S11 Fig**, adjusted FDR to match the expected total number of genes involved in the RLR pathway, see **Methods**). This compares to a genome-wide false discovery rate (i.e. when predicting genes randomly) of ~99%. Thus, the integrated RLR score increases the probability of correctly identifying novel RLR genes by a factor of 43 compared to random classification.

Because we used the same gene sets for calculating the RLR scores and estimating the performance of the resulting predictions (i.e. without systematic cross-validation), there exists a danger of circular reasoning. Therefore, we also carefully validated the quality of the results using various independent and external data sets. First, we examined the high RLR scores for genes that have a known function in innate immunity, but not in the RLR pathway, and therefore were not part of our training set. Components of other PRR signaling pathways (TLR, CLR, NLR, cytDNA) have lower scores than RLR genes, but much higher scores than the rest of the genome (**Fig 1B**). The same is true for genes functioning in other aspects of the innate immune response (**Fig 1B**). Of the 225 novel predictions (i.e. those genes that are not part of the training sets) in the top 354 (FDR of 57%, see above), 142 (~63%) are part of these innate immunity gene lists (**Fig 1C**). Thus, the majority of high-scoring genes with no known link to the RLR pathway in fact have a function in other PRR pathways or other parts of innate immunity, supporting the relevance of our predictions.

Second, we compared our predictions to six recent data sets that are relevant to the innate (antiviral) response but that were in no way part of the RLR score calculations. The overlap with the 354 top genes, excluding known RLR genes, is significantly larger than expected by chance for all these data sets (**Table 2**). For example, the top predictions include: (i) 19 of 45 (42%) interferon-stimulated genes with validated antiviral activity against e.g. HIV–1, HCV, yellow fever, West Nile or chikungunya virus [16], (ii) 27 proteins from a set of 241 (11%) that interact with the type I IFN protein network during pattern recognition, among which are five confirmed modulators of IFNβ expression and antiviral activity [12], (iii) nine tripartite motif (TRIM) family genes, five of which enhance RIG-I-induced activation of IFNβ, NFκB and ISRE (IFN-stimulated response element) promoters [17], and (iv) 38 human proteins interacting with innate immune-modulating viral open reading frames (viORFs) from 30 viruses [37]. (v) Furthermore, the type I IFN response has recently been proposed to play a role in antifungal immunity [38,39] and the top RLR predictions are strongly enriched for genes expressed in PBMCs stimulated with the fungal pathogen *Candida albicans*: almost half (39/89 = 44%) of these occur in our top predictions (*P* = 4.7 × 10^−47^, one-tailed Fisher’s exact test, **Table 2**). (vi) Finally, the overlap between our predictions and a genome-wide screen for regulators of RIG-I-mediated IFNβ production is, at only nine, marginal but significant (9/226 genes = 4%, *P* = 9.5 × 10^−3^, one-tailed Fisher’s exact test, **Table 2**) [14]. In summary, these diverse and independent experimental data support the validity of our integrated RLR score for predicting genes with a role in the innate antiviral response.

### RNAi validation screens confirm the high predictive value of the integrated RLR score

**Fig 2:**
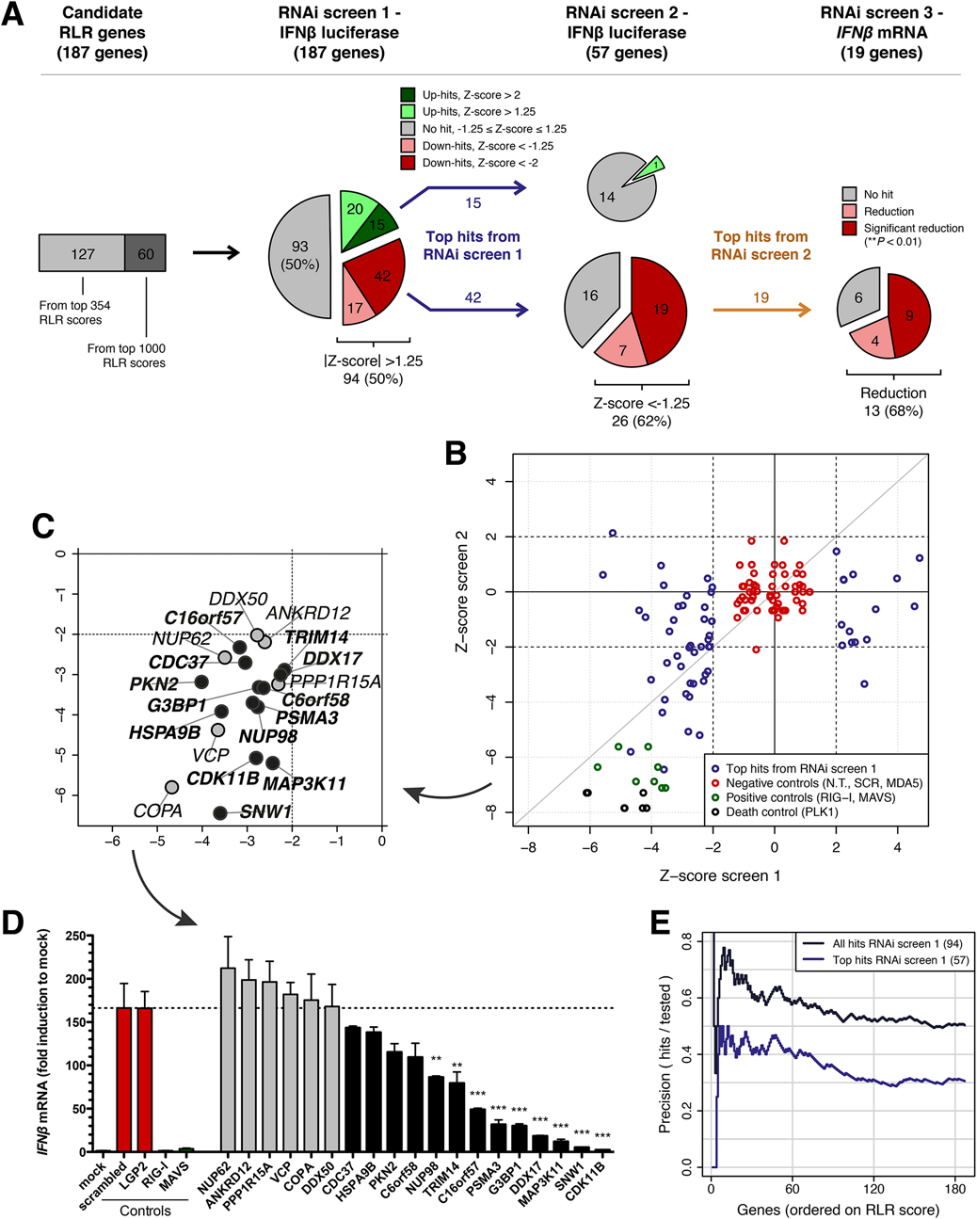
RNAi screens validate a role for the novel RLR candidates in RIG-I-mediated IFNβ induction. (**A**) Flow chart of the RNAi validation screens. 187 candidate RLR genes were screened for RIG-I pathway activity in three different RNAi screens. In screens 1 and 2, HeLa cells stably expressing an IFNβ promoter-controlled firefly luciferase (Fluc) reporter were stimulated with a 5’-ppp-containing RIG-I RNA ligand. The 57 hits (15 up, 42 down) with the largest effect on IFNβ induction upon siRNA knockdown in screen 1 (stringent Z-score <-2 or >2) were tested again in screen 2 with a different set of siRNAs. The 19 top hits from screen 2 were then picked for screen 3, which is similar to the first two screens except that it measures *IFNβ* mRNA levels using quantitative real-time qRT-PCR. (**B**) Correlation between the negative control-based robust Z-scores of RNAi screens 1 and 2. The 57 top hits with Z-scores <-2 or >2 in screen 1 were tested again in screen 2 (purple data points). N.T., non-transfected; SCR, scrambled. (**C**) Overview of the 19 novel RIG-I pathway genes with the largest effects on IFNβ induction in screens 1 and 2 (Z-score <-2 in both screens). Black data points correspond to genes whose knockdown also causes a reduction in *IFNβ* mRNA levels in screen 3. (**D**) RNAi screen 3. 13 of the 19 top hits from screens 1 and 2 also reduce RIG-I-mediated *IFNβ* mRNA production (black bars). Experiments were performed in triplicate (n = 3). Bars (mean±SEM) display the fold induction of *IFNβ* mRNA (corrected for actin mRNA levels) compared to the mock-treated control. Statistical significance was assessed by one-way analysis of variance (ANOVA) followed by Dunnett’s multiple comparison test, comparing the values for each of the 19 test genes to the combined negative control conditions (scrambled and LGP2, red bars). ** *P* < 0.01; *** *P* < 0.001. (**E**) Correlation between the *in silico* integrated RLR score and the probability of experimental confirmation in RNAi screen 1. The dark purple line represents all 94 hits with Z-score <-1.25 or >1.25; the light purple line represents the top 57 hits with Z-score <-2 or >2. The 187 experimentally tested genes were rank-ordered based on the RLR score and precision was calculated sequentially as the fraction of validated hits among all tested genes having a certain RLR score or higher.

To further determine the predictive power of our *in silico* predictions, we selected 187 candidate RLR genes for experimental validation (**S6**and **S8 Tables**). These include 127 high-confidence candidates from the top 354, which have not been previously linked to the RLR pathway, supplemented with 60 candidates we selected from the top 1000 predictions, mainly on the basis of limited functional characterization in general (**Fig 2A**). Importantly, candidates with a known role in RLR signaling, other branches of PRR pathways, or apoptosis were excluded as we were most interested in finding novel components of the RLR pathway.

For the selected candidates we performed a medium-throughput RNAi screen (RNAi screen 1) using HeLa cells stably expressing an IFNβ promoter-controlled firefly luciferase reporter (HeLa-IFNβ-Fluc). To activate the RLR pathway and induce Fluc reporter expression we used a known small 5’-ppp-containing RIG-I ligand [40]. This setup led to specific activation of RIG-I, as RIG-I or MAVS siRNA transfection, but not MDA5 or scrambled siRNAs, resulted in loss of reporter activity (**Figs 2B**, **S12**and **S13**). All negative controls (non-transfected, scrambled and MDA5 siRNAs) scored within 1.25 median absolute deviations of the plate normalized IFNβ induction levels (Z-score cutoff <-1.25 or >1.25, **Fig 2B**). At this cutoff, siRNA knockdown of 94 candidates (50% of all candidates tested) affected RIG-I-mediated IFNβ induction (**Figs 2A** and **S13A–S13D**and **S8 Table**). Among these, knockdown of 59 genes decreased RIG-I-mediated IFNβ induction (down-hits) and 35 genes increased IFNβ induction (up-hits). It is important to note that the experimental approach only activates the RIG-I branch of the RLR pathway and will not confirm predicted RLR candidates that regulate MDA5 activation and downstream signaling to MAVS. Thus, among the 93 non-confirmed candidates, there might still be novel regulators of the MDA5-mediated IFNβ induction pathway, which should be further investigated. Altogether, the integrated RLR score is clearly a strong and reliable predictor for novel regulators of the RIG-I pathway.

From the 94 confirmed hits, we picked the 57 top hits with the largest effect (stringent Z-score <-2 or >2) for a second RNAi screen using a different set of siRNAs (RNAi screen 2, **Fig 2A**). In this second RNAi screen, only a single up-hit (7% of 15 up-hits tested) showed a Z-score >1.25. Besides this hit, two negative control wells also had a Z-score >1.25 (**Figs 2B**, **S13E–S13H**), which suggests that the single confirmed up-hit might be unreliable. The poor reproducibility of the up-hits might be attributed to the screening approach. For instance, we used a large amount of 5’-ppp-containing RIG-I ligand (see **Methods**), leaving limited room for increased pathway activation. In contrast, the second RNAi screen confirmed 26 down-hits at Z-score <-1.25 (62% of the 42 down-hits tested). Of these, 19 genes (45% of tested down-hits) could be confirmed at a conservative Z-score <-2 (**Fig 2A, 2B and 2C**, **S8 Table**). Taken together, the two RNAi screens, guided by the predicted RLR candidates, have substantiated the validity of our approach and have revealed potential novel regulators of the RIG-I receptor pathway.

To gain further understanding of how the 19 top hits affect RIG-I-mediated IFNβ promoter activation, another RNAi screen was performed (RNAi screen 3). In contrast to the first two screens, here we did not use the IFNβ promoter-controlled Fluc reporter translation as readout, but we measured *IFNβ* mRNA levels using quantitative real-time (qRT)-PCR. As expected, knockdown of RIG-I and MAVS abrogated 5’-pppRNA-induced *IFNβ* mRNA transcription, while MDA5 knockdown [40] and LGP2 knockdown, which regulates only the MDA5-mediated *IFNβ* mRNA transcription, had no effect (**Fig 2D**). Of the 19 top hits from the first two RNAi screens, 13 genes (68%) in this third screen again showed a reduction in RIG-I pathway activation. Nine of these showed a significant reduction (*NUP98*, *TRIM14*, *C16orf57*, *PSMA3*, *G3BP1*, *DDX17*, *MAP3K11*, *SNW1*, *CDK11B*; *P* < 0.01, one-way ANOVA with Dunnett’s post hoc test; Fig 2A and 2D), suggesting that these gene products play a so far uncharacterized role in the RIG-I signaling pathway upstream of *IFNβ* mRNA transcription.

In summary, using RNAi-based screening methods we validated more than 50% of the tested candidates. To further assess the predictive power of the *in silico* integrated RLR score, we ranked the experimentally tested genes based on their RLR score and sequentially calculated the fraction of hits (either considering all 94 hits from RNAi screen 1, or only the 57 top hits) among all tested genes having a certain RLR score or higher (**Fig 2E**). Higher RLR scores were experimentally confirmed more often, indicating that these indeed correspond to more confident predictions. Further analysis revealed that there is no molecular signature that solely explains the predictions of the validated hits; rather the integrated score of the 10 molecular signatures is important (**S14 Fig**).

## Discussion

Knowledge about the constituents of biological systems and pathways is an essential step towards understanding their function in health and disease. In this study, we showed that existing biological data can be exploited to successfully identify novel components of a key intracellular defense pathway; the antiviral RIG-I-like receptor (RLR) pathway. The RLR pathway is important for detecting viral infections, and its dysfunction can increase susceptibility to infections with viruses [8] and fungi [39], but is also associated with autoimmunity [9,10]. We systematically investigated a large variety of genome-scale data for their ability to predict RLR pathway components, covering most of the important (gen)omics data types such as protein-protein interactions, gene (co-)expression, genetic interactions from RNAi screens, comparative genome analysis, and transcription regulation. In these data, we found five virus-based and five pathway-based molecular signatures of RLR pathway components, providing insight into the determinants of antiviral signaling and type I interferon production. Bayesian integration of the signatures led to the genome-wide prioritization of novel RLR pathway components. We subsequently validated the predictions by comparing them with various independent data sets and experimentally confirmed more than 50% of 187 selected novel RLR candidates for a role in RIG-I-stimulated IFNβ induction. These results reiterate the potential of computational assessment and combination of available biological data as a complementary approach to studies generating novel large-scale data sets.

### Identification of predictive signatures using a knowledge-based approach

To identify defining signatures of RLR genes in genomics data, we largely depended on current knowledge of the biology of the RLR system and its relationship with viruses. For example, since previous studies had shown that viral antagonism of specific RLR pathway components is prevalent [5,6], one of the first features we investigated, and indeed established, was that human-virus PPIs are a general theme for the RLR pathway as a whole. Similarly, guided by previous observations, we demonstrated that RLR genes conform to the tendency of immunity genes to evolve rapidly and commonly contain innate antiviral TF binding motifs, such as IRF and NFκB, in their promoters.

We also included several criteria that are effective for many different biological systems, but were specifically aimed at predicting novel RLR pathway genes in our case, such as the RLR co-expression calculations and RLR protein domain occurrences. We decided not to include associations based on text mining of published literature (e.g. co-mentioning of gene names in abstracts), because such approaches in our hands only enriched for genes already known to be involved in the RLR pathway and therefore compromised our ability to identify novel candidates. Finally, we settled on using a total of 10 molecular signatures that are relevant and predictive for the RLR system. Inclusion of additional data sets, generated for example by future experimental techniques, and substitution of existing data with novel and improved versions, will likely refine this data-driven definition of RLR genes over time and lead to updated Bayesian RLR probabilities that could further improve prediction accuracy.

A major challenge in our study arises from the fact that the RLR pathway is highly interconnected with other intracellular pathways, such as other innate PRR pathways (e.g. TLR and cytosolic DNA sensing), the stress response pathway, mitogen-activated protein kinase (MAPK) signaling cascades (e.g. TRAF2 and 6 lead to the p38 MAP kinases), and apoptosis (e.g. via CASP8 and 10) (**S1 Fig**) [2,12,41,42]. Although our approach for predicting novel RLR components relied on a well-defined set of genes known to make up the core of RLR signaling, the overlap with other systems was a potential confounding factor. For example, most molecular signatures of RLR genes identified here, especially the virus-based properties such as PPIs with viruses, rapid evolution, and differential expression during infection, could also apply to genes involved in other aspects of antiviral immunity. Nevertheless, combination of the right signatures achieved reasonable specificity for RLR genes (**Fig 1**). Thus, we have extended an approach previously used for identifying components of membrane-enclosed organelles such as the mitochondrion [19] and showed that it is also possible to capture the complexity of a diverse and interconnected intracellular signaling pathway. The presented approach for identifying predictive signatures, followed by Bayesian integration, could potentially be applied to any cellular system.

### Contributions of the individual signatures to the RLR predictions

Using the sets of known RLR and non-RLR genes, we could systematically assess the relative quality of the individual data sets for predicting novel RLR genes. Indeed the 10 molecular signatures have different predictive values as shown by the likelihood ratio scores (**Fig 1A**, **Tables 1**and **S1**), and thus contribute with different weights to the integrated Bayesian RLR score. The data types with the strongest predictive value include NFκB activation mediators, RLR pathway protein domains, and both PPI signatures (PPIs within the RLR pathway and PPIs between human and viral proteins). In contrast to our expectations, antiviral host factors identified in high-throughput RNAi experiments had a relatively small contribution. Besides raw predictive ability, we also considered the coverage of the data sets. For example, there are only few (<200) NFκB activation mediators and antiviral host factors, while the data sets on RLR co-expression, viral miRNA targets, and innate antiviral TF binding motifs identified many more genes (>4,000). Integration of all data sets with their varying coverage and predictive value into a single RLR score resulted in a classifier that is superior to the individual data sets (**Fig 1D**). This is underscored by the observation that the individual signatures by themselves are unable to explain the predictions for the experimentally validated RLR candidates, and only the integrated RLR score explains all validated genes (**Figs 2E** and **S14**).

### Independent studies validate additional RLR candidates

Aside from our own experimental validation strategies, recent independent studies have confirmed a role for 15 of our predicted RLR candidates in the RLR pathway during viral infection (**Table 3**). Most of these publications appeared during the course of our study, and thus were not part of the knowledge or data used for predicting novel RLR genes. For example, TRIM14 (RLR rank 491) has been demonstrated to interact with MAVS leading to activation of IRF3 and NFκB via IKKγ (NEMO) [43]. Indeed, our predictions marked TRIM14 as a strong candidate RLR gene and all our RNAi screens confirmed it as a component required for optimal RIG-I signaling (**Fig 2**). Two additional high-confidence RLR predictions for which we validated an effect in all three RNAi screens have recently been validated externally as well: G3BP1 [44] and CDC37 [13].

**Table 3:** Validations of our predicted RLR candidates by independent studies.

Gene symbol	Gene description	RLR rank	Described function	References	Type of regulation (literature) ^a^	Type of regulation (our RNAi screens) ^b^
*CSNK2A1*	Casein kinase II subunit alpha	45	The casein kinase II complex inhibits the RIG-I-mediated antiviral response through phosphorylation of RIG-I	[93]	**-**	0^c^
*TRIM38*	Tripartite motif-containing protein 38	56	Negative regulator of RIG-I-mediated IFNβ production by targeting AZI2 (NAP1) for degradation	[94]	**-**	**-**
*RNF11*	RING finger protein 11	78	Interacts with TBK1 and IKBKE (IKKε) to block TRAF3 interaction and restrict IRF3 activation	[95]	**-**	
*SMAD3*	SMAD family member 3	100	Regulates dsRNA-induced transcriptional activation of IRF7 at the IFNβ promoter	[96]	**+**	0
*UBE2D1*	Ubiquitin-conjugating enzyme E2 D1	139	This Ubc5 E2 ligase is required for viral activation of IRF3 and MAVS by RIG-I	[97]	**+**	0
*CDC37*	Hsp90 co-chaperone Cdc37 (cell division cycle 37)	165	Regulates stability of TBK1 via Hsp90, allowing for induction of IFNβ in response to DNA viral and retroviral infections	[13]	**+**	**+**
*RNF114*	RING finger protein 114	181	Enhancer of dsRNA-induced production of type I IFN through positive feedback regulation	[45]	**+**	**-**
*SRPK1*	Serine/threonine-protein kinase SRPK1	235	Enhancer of RIG-I-dependent IFNβ and IFNλ1 promoter activation during Sendai virus infection, possibly via IRF3/7 phosphorylation	[98]	**+**	**-**
*CSNK2A2*	Casein kinase II subunit alpha prime	249	The casein kinase II complex inhibits the RIG-I-mediated antiviral response through phosphorylation of RIG-I	[93]	**-**	0^c^
*G3BP1*	GTPase-activating protein-binding protein 1	282	Functions in the formation of stress granules, which act as RLR signaling platforms that in some cases enhance IFN induction	[44]	**+**	**+**
*UBE2I*	SUMO-conjugating enzyme UBC9	284	Enhances RIG-I and MDA5 SUMOylation, which correlates with increased IFNβ expression and repressed virus replication	[99,100]	**+**	
*SUMO1*	Small ubiquitin-related modifier 1	326	IRF3/7 SUMOylation down-regulates IFN production; RIG-I/MDA5 SUMOylation correlates with increased IFNβ expression	[99–101]	**- / +**	
*PPP1R15A*	Protein phosphatase 1 regulatory subunit 15A	389	Required for IFNβ production induced by dsRNA and chikungunya virus in mouse; expression depends on PKR activation	[102]	**+**	**+**
*TRIM14*	Tripartite motif-containing protein 14	491	Interacts with MAVS upon viral infection, thereby recruiting IKKγ (NEMO), which leads to activation of IRF3 and NFκB	[43]	**+**	**+**
*DDX60*	DEAD box protein 60	616	Promotes virus-induced, RLR-mediated type I IFN expression and increases binding of RIG-I to dsRNA	[103]	**+**	
				**Total:**	15	7 hits (out of 11)

^a^ '+': positive regulator (expected decrease in IFNβ induction upon knockdown). '-': negative regulator (expected increase in IFNβ induction upon knockdown).

^b^ Annotated cells (‘+’, ‘-’, ‘0’) indicate 11 candidate RLR genes that were tested in RNAi screen 1. ‘+’: down-hits from RNAi screen 1 (decreased RIG-I-mediated IFNβ induction upon knockdown, Z-score <-1.25). ‘-’: up-hits from RNAi screen 1 (increased RIG-I-mediated IFNβ induction upon knockdown, Z-score >1.25). ‘0’: no hit in RNAi screen 1, or inconsistent effect across RNAi screens 1 and 2 (*CSNK2A1* and *CSNK2A2*, ^c^).

Of the 15 genes recently described in the literature, 11 were part of the candidate RLR genes tested in our RNAi screens (**Table 3**). Of these, seven genes affected RIG-I-mediated IFNβ induction in RNAi screen 1 (Z-score <-1.25 or >1.25) and showed a consistent effect in RNAi screen 2. Therefore, our experimental screening condition appears to detect these described RIG-I pathway regulators with a sensitivity of ~64% (7/11). Furthermore, four out of four down-hits from our experiments (i.e. genes that decreased IFNβ induction when knocked down, hence positive regulators) that have been described in the literature were indeed described as positive regulators of RIG-I signaling (**Table 3**). Given that our experimental approach detected most, but not all, of the published RIG-I regulators, a substantial number of our predicted RLR candidates not validated by our RNAi screens might still play a role in for example a different cell type, downstream of type I IFN production, or regulate the pathway via MDA5/LGP2 activation. For example, RNF114 (RLR rank 181, Z-score RNAi screen 1 = 1.68, **Table 3**) is an ISG and therefore needs to be up-regulated via a positive feedback loop to fully contribute to RLR pathway stimulation [45]. This gene was not confirmed in all RNAi screens, perhaps because the time of RIG-I stimulation in our screens (6 hours) was simply too short. Similar biological reasons could limit the detection of an effect for other genes as well. Therefore we conclude that the hits identified in our RNAi validation experiments may be a conservative estimate of the number of correct RLR predictions.

### Novel RIG-I pathway components DDX17 and SNW1 could regulate activation of transcription factors NFκB and IRF3

We identified 13 novel RIG-I pathway regulators that reduced IFNβ induction in all three RNAi screens (**Fig 2**). These include cell cycle gene *CDK11B*, heat shock protein *HSPA9B*, MAP kinase *MAP3K11*, proteasome subunit *PSMA3*, nucleoporin *NUP98* [46], and the recently identified RLR regulators *CDC37* [13], *G3BP1* [44] and *TRIM14* [43] (**Table 3**). The remaining five genes, *DDX17* (DEAD box helicase 17), *C6orf58*, *C16orf57* (*USB1*, U6 snRNA biogenesis 1), *PKN2* (serine/threonine protein kinase N2), and *SNW1* (SNW domain containing 1), are overall least characterized. To obtain a first suggestion about how these genes might regulate RLR signaling, we searched for connections with the known human and viral protein interaction networks. Next, we discuss the reported interactions of DDX17 and SNW1 with the RLR pathway.

**Fig 3:**
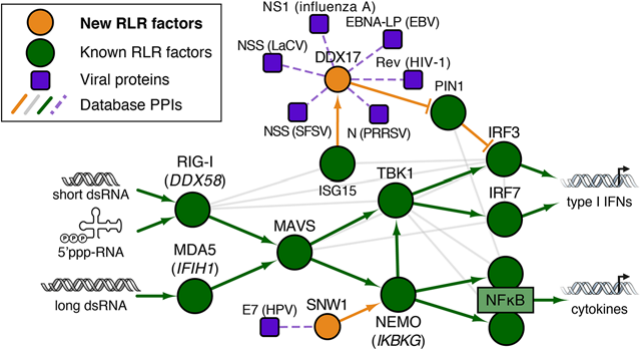
Human and viral protein interaction networks connecting the known RLR pathway with the newly identified RIG-I factors DDX17 and SNW1. Human proteins are represented by circles, viral proteins by rounded rectangles (purple nodes). Green nodes represent known components of the RLR pathway. Orange nodes (DDX17 and SNW1) are novel RIG-I pathway components discovered in our study, which are connected to the RLR network through interactions with the green nodes. Edges between human proteins represent physical interactions (both low- and high-throughput) obtained from BioGRID Release 3.3 [54]. Interactions between human and viral proteins were obtained from the PHISTO database (29 Sep. 2014) [28]. See **S1 Fig** for a more complete representation of the RLR pathway containing the curated set of 49 known RLR genes. LaCV, La Crosse virus; EBV, Epstein-Barr virus; SFSV, Sandfly fever Sicilian virus; PRRSV, Porcine reproductive and respiratory syndrome virus; HPV, Human papillomavirus.

DEAD box RNA helicase DDX17 was recently found to bind Rift Valley fever virus RNA and restrict viral replication in an interferon-independent manner [47]. Our data now suggest a role for DDX17 in RIG-I-mediated IFNβ production as well. DDX17 has reported protein interactions with two other RIG-I regulators identified in our study: CDC37 and CSNK2A1. Interestingly, DDX17 also interacts with the peptidylprolyl cis/trans isomerase PIN1 [48], which inhibits RIG-I-mediated IFNβ production by inducing degradation of IRF3 [49]. Furthermore, DDX17 was present among a set of ISG15-modified (ISGylated) proteins in HeLa cells treated with IFNβ [50]. Thus, DDX17 could function in IRF3 activation by acting as a negative regulator of PIN1 and might be regulated by ISGylation (**Fig 3**). Lastly, DDX17 seems to be a preferred target of viral interference, having reported interactions with six different viruses (e.g. HIV–1 Rev and influenza virus A NS1, **Fig 3**).

SNW1 is an intrinsically disordered protein [51,52] that interacts with two other newly identified RLR regulators from our study, namely PKN2 [53] and C16orf57 [54]. SNW1 also interacts with the IKBKG (NEMO) protein [55], which is required for NFκB and IRF3 activation [56]. Given that our data shows that knockdown of SNW1 reduces IFNβ induction, SNW1 could be involved in NEMO regulation and thereby contribute to activation of the RLR pathway TFs, NFκB and IRF3 (**Fig 3**). The fact that SNW1 was also identified in a siRNA screen for mediators of virus-induced NFκB activation [34] strengthens this hypothesis. Further studies should be conducted to resolve the precise mode-of-action.

### The genome-wide prioritization of RLR pathway components is a new resource for innate antiviral immunity research

We have validated the integrated RLR score with various experimental, literature and computational approaches. Our confirmations of a substantial fraction of the predicted RLR genes suggest the value of the prioritized list as a whole. The genome-wide prioritization of RLR pathway components is available in **S6 Table** and at http://rlr.cmbi.umcn.nl/, and can be used as a resource in several ways. For example, it can serve in the evaluation of data sets relevant to the innate antiviral and antifungal responses (**Table 2**). Many labs routinely consult internal data sets to decide which genes to study further. Comparison of such lists with for example high-scoring RLR candidates could provide insights into the quality of individual data sets for identifying antiviral genes and provide complementary hints about which genes could be important. Finally, the RLR resource could be used for prioritizing genetic variants in patients suffering from severe susceptibility to viral infections or inflammatory disorders caused by inappropriate production of type I interferons.

### Conclusions

In this work, we have combined integrative genomics with experiments to discover 10 molecular signatures of a cellular signaling system that is central to human infectious disease: the innate antiviral RIG-I-like receptor (RLR) pathway. The described signatures span multiple layers of genomics data and provide new insights into the regulation of virus detection and immune signaling. Probabilistic integration of the data resulted in a confident genome-wide ranking of candidate RLR pathway genes. RNAi validation experiments confirmed 94 of 187 novel RLR candidates tested, including 13 novel factors with strong effects on antiviral signaling. These results, together with independent computational and literature-based confirmation, demonstrated the validity and high accuracy of our approach. Our study expands the collection of known antiviral genes, opening up new avenues for research into innate antiviral immunity.

## Methods

### Human reference proteome and mapping

All data sets were calculated for and mapped to a reviewed reference set of 20,245 human proteins from UniProtKB/Swiss-Prot, release 2011_11 [57]. This set consists of one manually annotated record for each validated protein-coding gene. Gene/protein identifier mapping was performed using a mapping table from the same UniProt release. Ambiguously mapped identifiers were curated manually.

### Molecular signature data sets

To systematically define RLR pathway components, we mined genome-scale data from a wide variety of sources. The data describe different aspects of the biology of the pathway; from the DNA to the protein level, highlighting evolutionary processes, virus-host interactions, sequence families, etc. We finally settled on 10 data sets that collectively distinguish RLR pathway components from other genes (see **Table 1**for an overview and brief descriptions):

### Virus-based signatures

#### Positive selection in primates

George *et al*. [23] calculated dN/dS-based likelihoods for recurrent positive selection across the exomes of seven primates (human, chimpanzee, orangutan, rhesus macaque, vervet, colobus monkey, tamarin). Maximum likelihood analysis of the nucleotide alignments of ~15,000 genes identified 930 genes with evidence for positive selection at *P* < 0.05. We grouped the genes according to these positive selection *P* values.

#### Protein-protein interactions (PPI) with viruses

Recent years have seen a surge of studies reporting interactions between viral and human proteins, both small- and large-scale. First, we collected all known virus-human PPIs from five specialized resources: PIG (9 Sep. 2011) [58], HPIDB (9 Sep. 2011) [59], VirHostNet 1.0 (24 Oct. 2011) [60], VirusMINT (6 Dec. 2011) [61], and PHISTO (25 Jan. 2012) [28]. These data were then combined to determine all human proteins for which an interaction was reported with at least one virus (**S2 Fig**). Of note, the interactions reported by Pichlmair *et al*. [37] are not part of the final data set of virus-human PPIs and thus could be used for independent validation of the predictions (**Table 2**).

#### Viral miRNA target

Likely human target genes of viral miRNAs were determined in three steps. First, from the vHoT database we collected transcriptome-wide TargetScan (v5.0) predictions for the binding of 128 miRNAs from nine, mostly DNA viruses to the 3'UTRs of human mRNAs (**S2 Table**) [30]. More negative scores are associated with more favorable binding site predictions. Second, because a single human transcript may be targeted (i) at multiple sites by a single miRNA and (ii) multiple times by different miRNAs, for each transcript we summed the prediction scores for all predicted target sites of all viral miRNAs. The resulting score (‘viral miRNA targeting score’) represents the overall likelihood that viruses target that mRNA. Third, the final score per gene was defined as the most negative score across its transcripts.

#### Differential expression upon infection

Human lung alveolar type II cells (A549) were cultured and exposed to four live respiratory viruses as described previously [62]: respiratory syncytial virus (RSV), human metapneumovirus (hMPV), parainfluenza virus type 3 (PIV), and measles virus (MV). RNA was isolated at 6, 12, and 24h post infection, as well as from mock-infected (medium without virus) control cells. Gene expression was then measured using the Affymetrix U133 plus 2.0 GeneChip platform and infection conditions were compared to uninfected cells. Data were log_2_-transformed and normalized by VSN [63]. Statistically significant differential expression for each probe set (54,675 in total) was assessed using *limma* [64] and expressed as the fold change in expression between infected and uninfected conditions (FDR cutoff of 0.05). Genes represented on the microarray platform by multiple probe sets were summarized by the median differential expression across their probe sets. From the transcriptomics data we calculated for each gene (20,190 genes in total) the maximum absolute (i.e. considering both up- and down-regulation) change in expression across all time points and viruses, compared to uninfected cells. The column ‘Differential expression’ of **S6 Table** contains the processed gene expression data. A full analysis of these experiments will be described in a later publication.

220 and five genes were significantly up- and down-regulated in at least one infection condition respectively (>1.5 and <-1.5 log_2_ fold expression changes). A total of 1,761 genes showed maximum absolute differential expression >0.5. Infection with hMPV induced maximal expression changes for the majority of genes (63% of the 1,761 genes with maximal absolute fold change >0.5), followed by RSV (29%). In comparison, PIV (4%) and MV (3%) caused less pronounced expression changes. Indeed, the expression profiles confirm these trends (**S3A Fig**). Furthermore, most genes tend to be increasingly up- or down-regulated during the course of infection (S3A and S3B Fig), with the distribution of expression changes becoming more extreme going from 6h (~5% of the 1,761 genes with maximal absolute fold change >0.5), to 12h (~14%), to 24h (~80%).

#### Antiviral host factor

We collected data from large-scale forward genetics screens aimed at identifying human genes involved in viral replication. Most studies focus on factors that *reduce* viral replication when inactivated, as these are often most abundant and represent candidate drug targets for infection treatment. However, these screens can also identify antiviral host factors, or host restriction factors, that inhibit virus replication (i.e. *increase* viral replication when inactivated). We collected the results from seven RNAi studies that investigated infection of human cells with a variety of viruses. These screens together reported a total of 173 unique antiviral host factors (**S3 Table**).

### Pathway-based signatures

#### Co-expression with RLR pathway

Functionally related genes tend to share expression patterns, i.e. be co-expressed. We employed an expression data integration method that weighs expression data sets for co-expression within a specific biological system [32]. From the NCBI gene expression omnibus database (GEO) [65] we obtained a collection of 465 publicly available human microarray data sets (~10,000 individual measurements). Each set of mRNA expression measurements was then assessed for its potential to find novel RIG-I-like receptor pathway genes by determining the coherence of expression of the 49 known RLR genes. That is, for each data set we ask whether known RLR genes behave similarly in terms of their expression, being up- or down-regulated together in the same microarray measurement. Sets of expression measurements in which known RLR genes show coherent expression receive a high weight, and will contribute more to the co-expression calculation than experiments with less coherent expression of known RLR genes. These weights are then used to calculate an integrated score for each gene in the human genome, according to how much its expression profile correlates with that of the RLR genes across the expression data sets (**S4 Fig**).

As the co-expression method was trained with the aim of retrieving RLR genes with high reliability, we also assessed its ability to retrieve RLR genes in leave-one-out cross-validation analysis. For that, we calculated the weighted co-expression 49 times, leaving out one of the 49 RLR genes in each fold (so that the whole set was left out exactly once), and determined the co-expression rank of the RLR gene that was left out. For the other gene sets (i.e. covering all genes except the RLR genes), we averaged the co-expression ranks across the 49 cross-validation runs. **S4D Fig** shows the recall (also known as sensitivity) of various gene sets at each rank cutoff in the cross-validation: genes were rank-ordered based on the RLR co-expression cross-validation rank and recall for each gene set was calculated sequentially as the fraction of genes among all genes in the set having a certain rank or higher.

#### RLR pathway protein domain

Domain organizations for all human proteins in SwissProt were obtained from the Pfam database (release 26.0; SwissPfam) [66]. We calculated statistical over-representation of domains occurring in the 49 known components of the RLR pathway compared to the background of all human proteins using the Fisher’s exact test. Enrichment *P* values were corrected for testing multiple domains (40 in total) using the Benjamini-Hochberg (BH) false discovery procedure and judged to be significant at a significance level of 1% (**S4 Table**). Finally, we determined a set of proteins that contain one or more such enriched ‘RLR domains’.

#### Innate antiviral transcription factor (TF) binding motifs

Conserved TF binding sites in human were obtained from a comparative analysis of 29 genomes of placental mammals [33]. In this study, TF regulatory motif instances (putative TF binding sites) were detected across the human genome and assigned a likelihood based on conservation across the 29 mammals: for each motif match in human, the smallest phylogenetic subtree was calculated that contains the human motif and aligned motifs in other species [67]. To identify putative transcription regulators of a gene, we extracted conserved TF binding sites in promoter regions, which were defined as 4 kilobase (kb) windows centered (i.e. 2kb upstream and 2kb downstream) at all annotated transcription start sites of the gene [68]. We then searched for genes containing conserved motifs associated with four key innate antiviral transcription factors (IRF, AP–1, NFκB, and STAT; **S5 Table**). Finally, we grouped all genes by the number of distinct motifs found: none, one, two, three or four.

#### NFκB activation mediator

Gewurz *et al*. undertook a genome-wide siRNA screen for NFκB pathway components [34]. They studied HEK293 cells with a stably integrated NFκB GFP reporter and inducible expression of Epstein-Barr virus latent membrane protein (LMP1), which activates NFκB. 155 LMP1 activation pathway components were identified, many of which are also important for IL–1β-, or TNFα-mediated NFκB activation. We obtained these hits and mapped them to 154 protein identifiers.

#### RLR pathway PPI

Human protein-protein interactions were obtained from the PINA database (release 28 Jun. 2011), which contained ~75,000 PPIs from six major resources [35]. We took all interactions involving the 49 known RLR pathway proteins and counted how many interactions each protein is involved in, thus obtaining 1,750 proteins with at least one RLR interaction. Of note, the interactions reported by Li *et al*. [12] are not part of the final data set of RLR pathway PPIs and thus could be used for independent validation of the predictions (**Table 2**). We also assessed the cohesiveness of the RLR PPI network by calculating physical interaction enrichment scores, as described in [36].

### Training sets

We assessed the capability of individual data sets to predict novel RLR genes using two ‘gold standard’ training sets:

#### Positive gold standard

We used a curated standard of 49 genes that are well characterized to play a role in the RLR pathway and make up its core (‘RLR genes’, all of which are depicted in **S1 Fig**). This set is based mainly on the KEGG map [69] of the RLR pathway and taken from InnateDB (27 Mar. 2012) [21]. We focused on intracellular components, hence excluding the interferons and proinflammatory cytokines that are induced by the pathway.

#### Negative gold standard

To represent genes that are unlikely to play a role in RLR signaling, we constructed a set of 5,818 genes from seven functional categories generally unrelated to the innate antiviral response (‘non-RLR genes’, **S6 Table**).

1. Housekeeping genes. These are typically defined as genes showing constitutive and constant expression in 'all' tissues. We collected housekeeping genes from five different studies [70–74], and included 1458 genes that were reported in at least three studies.
2. Ribosomal subunits. 134 human ribosomal (cytoplasmic and mitochondrial) proteins from [75].
3. Transmembrane transporters. 986 confirmed and predicted cytoplasmic membrane transporters and membrane channels from [76].
  From QuickGO (6 Feb. 2012), we obtained human genes annotated with various gene ontology terms and their child terms [77], considering only annotations supported by experimental evidence codes (IMP, IGI, IPI, IDA, IEP, EXP):
4. Mitoplast localization. 559 genes with contributions to or co-localization with the mitochondrial matrix (GO:0005759) or mitochondrial inner membrane (GO:0005743). We did not include the inner membrane space and outer membrane, which is critical for RLR signal transduction through MAVS.
5. Metabolism. Genes with annotation ‘metabolic process’ (GO:0008152), excluding those annotated with the child term ‘protein phosphorylation’ (GO:0006468); 2243 genes.
6. Neurological functions. 1497 genes from GO term ‘neurological system process’ (GO:0050877).
7. Embryonic development. 775 genes from GO term ‘embryo development’ (GO: 0009790).

We removed genes from the negative set that are known RLR genes, components of other PRR signaling pathways (TLR, CLR, NLR, cytDNA), or other innate immunity genes (see below). The resulting negative set is a good reflection of the rest of the genome in terms of the distributions of the various molecular signatures and RLR integration scores (**Fig 1B**). Furthermore, given its size and the diversity of genes included, it is reasonable to expect a number of ‘non-RLR genes’ with high RLR scores. These should be considered as inappropriately included in the negative set and are therefore still candidate RLR genes.

### Other PRR pathway and other innate immunity gene sets

Two additional curated sets of genes were used in our study (**S6 Table**). The first consists of 153 genes with a known function (i.e. receptors, signaling components, etc.) in four PRR signaling pathways; the Toll-like receptor (TLR), C-type lectin receptor (CLR), NOD-like receptor (NLR), and cytosolic DNA sensing (cytDNA) pathways, but not in the RLR pathway. TLR, NLR and cytDNA components were obtained from InnateDB (27 Mar. 2012). We curated a list of 34 CLR pathway components, based mainly on [78]. The combined PRR pathway gene set was supplemented with several key proteins involved in virus-host interactions. As with the set of RLR genes, cytokines and other secreted proteins were excluded. The second list (‘other innate immunity genes’) consists of 803 genes with curated annotations from InnateDB (12 Jan. 2012) for a function in other aspects of the innate immune response, excluding RLR and other PRR signaling pathway genes.

### Naive Bayesian integration

Individual (genomics) data sets contain important information about the make-up of cellular systems and pathways, but often have limited coverage and introduce data type-specific noise. Combination of multiple heterogeneous types of data, each approaching the characterization of a molecular system from a different angle, therefore has the potential to provide a more complete definition of the system and could have high power for predicting novel components involved.

We employed a naive Bayesian framework to facilitate direct comparison and weighing of many data sets describing properties of RIG-I-like receptor pathway components and integrate those data sets that were suitable into a single probabilistic score for each gene. Bayesian integration is well suited to combining evidence from dissimilar types of information and readily accommodates missing data [18–20]. Furthermore, this approach inherently weighs data sets based on their predictive value (i.e. their ability to separate known positives and negatives, **Fig 1A** and **S1 Table**) so that better data contribute more to the predictions. Indeed, integration enriches for RLR genes and depletes false positive, non-RLR genes (**S5**and **S9 Figs**).

#### Calculation of the RLR score

For any given gene in the human genome, we can calculate the conditional probability that the gene is involved in the RLR pathway given the observed evidence in the 10 molecular signature data sets. More precisely, we calculated the posterior odds, defined as the ratio of the probability that the gene in an RLR gene versus the probability that the gene is not an RLR gene:

$$O_{posterior}=\frac{P(RLR\;gene|D_{1}\ldots \;D_{10})}{P(non-RLR\;gene|D_{1}\ldots \;D_{10})}$$

As this equation cannot be calculated directly, we approximate the ‘reverse’ likelihood ratio *L* that a certain combination of values for the 10 data sets are observed, given the distribution of known RLR and non-RLR genes (i.e. the positive and negative training genes) across the data:

$$L(D_{1}\ldots \;D_{10})=\frac{P(D_{1}\ldots \;D_{10}|RLR\;gene)}{P(D_{1}\ldots \;D_{10}|non-RLR\;gene)}$$

These two equations are related by Bayes’ theorem though the prior odds: the ratio of probabilities that any gene in the human genome is an RLR gene versus a non-RLR gene, prior to the use of information from our data sets. The prior odds can be calculated from the estimated total number of genes involved in the RLR pathway (see below).

$$O_{posterior}=O_{prior}\cdot L(D_{1}\ldots \;D_{10})$$

$$\frac{P(RLR\;gene|D_{1}\ldots \;D_{10})}{P(non-RLR\;gene|D_{1}\ldots \;D_{10})}=\frac{P(RLR\;gene)}{P(non-RLR\;gene)}\cdot \frac{P(D_{1}\ldots \;D_{10}|RLR\;gene)}{P(D_{1}\ldots \;D_{10}|non-RLR\;gene)}$$

An assumption of the *naive* Bayesian approach is that the individual sources of evidence are independent of each other. Although this is rarely completely the case with genomics data, limited violations of the independence assumption still lead to effective predictions (see below). Under the independence assumption, *L* can be simplified and calculated as the product of the likelihood ratios of the individual data sets:

$$L(D_{1}\ldots \;D_{10})=\prod_{i=1}^{10}\frac{P(D_{i}|RLR\;gene)}{P(D_{i}|non-RLR\;gene)}$$

We calculated these likelihood ratio scores for individual data sets (**Tables 1**and **S1**) directly from the contingency tables relating the positive and negative training genes to the data values binned into discrete intervals, asking: “What is the probably that a (non-)RLR gene has a value within a certain range in the data”? The bar plots in **Fig 1A** represent these contingency tables; likelihood ratio scores for each bin are defined as the ratios of the green versus red bars. Of note, as not all data sets contain values for all genes (e.g. genes can be missing from microarray platforms, were not tested in siRNA screens, etc.), we separated genes that *were* tested but show no effect from genes that were not tested. That is, we assigned no scores to bins that represent genes missing from the data entirely.

Having obtained the scores for the individual data sets and the prior odds, we then calculated the posterior odds that any gene is involved in the RLR pathway given its values in the data:

$$\frac{P(RLR\;gene|D_{1}\ldots \;D_{10})}{P(non-RLR\;gene|D_{1}\ldots \;D_{10})}=\frac{P(RLR\;gene)}{P(non-RLR\;gene)}\cdot \prod_{i=1}^{10}\frac{P(D_{i}|RLR\;gene)}{P(D_{i}|non-RLR\;gene)}$$

Finally, we obtained the ‘RLR score’ (**S6 Table** or http://rlr.cmbi.umcn.nl/) by log_2_ transformation of the individual terms in order to create an additive score:

$$RLR\;score={\text{log}}_{2}\left(\frac{P(RLR\;gene|D_{1}\ldots \;D_{10})}{P(non-RLR\;gene|D_{1}\ldots \;D_{10})}\right)={\text{log}}_{2}\left(\frac{P(RLR\;gene)}{P(non-RLR\;gene)}\right)+\sum_{i=1}^{10}{\text{log}}_{2}\left(\frac{P(D_{i}|RLR\;gene)}{P(D_{i}|non-RLR\;gene)}\right)$$

Taken together, the RLR score represents a Bayesian posterior probability, which depends on the positive and negative gold standard genes, the data sets used for the predictions, and the prior expected number of positive and negative genes in the genome. Although the RLR score may change for different priors (see below), the relative RLR ranks remain the same as these only depend on the gold standards and the data. Thus, the relative ranking of genes as captured in the RLR rank is most informative.

#### Conditional independence

Although violations of the independence assumption can lead to over-estimation of the likelihood scores, previous work has shown naive integration of genomics data to be effective for predicting novel genes involved in a molecular system [19,20]. Assessment of the pairwise correlations between the 10 genomics data sets used for predicting RLR genes suggests that they are largely complementary (**S10 Fig**). Several data sets have higher pairwise correlations, such as ‘PPI with viruses’ and ‘Innate antiviral TFs’. However, these features describe different molecular processes, namely protein-protein interactions between viral and human proteins and the presence of specific TF binding motifs, and hence can be considered largely independent in molecular terms.

#### Performance estimates

The performance of each of the 10 individual data types, as well as the integrated RLR score, for predicting RLR genes was evaluated using the positive and negative training sets. Based on these sets of known (non-)RLR genes, we calculated for each RLR score threshold (where genes with scores equal or higher than the threshold are predicted positives, i.e. predicted RLR genes, and genes with lower scores are predicted negatives, i.e. predicted non-RLR genes) the number of predictions that are:

- true positive (TP, number of positive training genes predicted as positive)
- false positive (FP, number of negative training genes predicted as positive)
- true negative (TN, number of negative training genes predicted as negative)
- false negative (FN, number of positive training genes predicted as negative)

These were then used to calculate several performance measures:

- $Sensitivity\;(SN)=\frac{TP}{TP+FN}$, fraction of positive training genes correctly predicted as positive (**Fig 1D**)
- $Specificity\;(SP)=\frac{TN}{FP+TN}$, fraction of negative training genes correctly predicted as negative (**Fig 1D**)
- $False\;Discovery\;Rate\;(FDR)=\frac{FP}{TP+FP}$, fraction of positive predictions that are false (i.e. that are negative training genes)

Calculation of the FDR depends on both the positive and negative gold standard genes. As the sizes of these training sets do not accurately reflect the expected numbers of RLR and non-RLR genes in the genome (prior probabilities, see below), we corrected the FDR to get an unbiased estimate using the following equation [19] (**S11 Fig**):

$${FDR}_{corrected}=\frac{1-SP}{1-SP+SN\cdot O_{prior}}$$

#### Prior estimation of the number genes involved in the RLR pathway

Determination of the probability of finding a gene in the genome with a role in the RLR pathway, prior to the use of additional information, requires an estimation of the expected total number of RLR genes. We estimated this at 300; six times the number of currently known RLR genes in the positive training set. The prior odds then become ~1.5%:

$$O_{prior}=\frac{P(RLR\;gene)}{P(non-RLR\;gene)}=\frac{P(RLR\;gene)}{1-P(RLR\;gene)}=\frac{\frac{300}{20,245}}{\frac{\left(20,245-300\right)}{20,245}}\approx 0.015$$

The prior odds influence the absolute RLR score and the corrected false discovery rate. Importantly, however, the overall ranking of genes does not depend on the estimated number of RLR genes. To assess the impact of the prior on the RLR score and false discovery rate, we re-calculated these measures using lower (75), medium (200) and upper (1000) bound estimates for the number of RLR genes (**S9 Table**). These results suggest maximum and minimum FDRs of 84% and 28% at rank 354 (compared to an FDR of 57% when using a prior of 300).

#### Separate assessment of co-expression, protein domain, and RLR pathway PPI signatures

As described before, a positive gold standard of 49 known RLR pathway genes was used for calculating the likelihood scores for individual data sets. However, three molecular signatures (co-expression, protein domain and RLR pathway PPI) originate directly from calculations based on this same set of RLR genes. To avoid circularity, we assessed the performance (sensitivity, specificity) and likelihood ratio scores of these data sets using a different, independent positive training set: components of other PRR signaling pathways (TLR, CLR, NLR, cytDNA, see above). This approach prevented over-estimation of the predictive ability of these data sets and ensured that the likelihood scores of all molecular signatures are in the same range.

### RNAi validation screens for RIG-I pathway activity

#### Cells and RIG-I ligand

HeLa-R19 cells stably expressing Firefly luciferase under control of the IFNβ (*IFNB1*) gene promoter were generated using the pIFNβ-Fluc-NeoR plasmid, which was kindly provided by Wendy Barclay [79]. Single cell clones were selected under G418 selection, and a mixed population of two positive clones was used for the screens. Cells were maintained in DMEM supplemented with 10% FCS in a humidified incubator with 5% CO2. As RIG-I ligand, we used 5’-ppp cloverleaf (CL) from coxsackievirus B3 (CVB3) sequence, a 90 nt ssRNA carrying a 5’ triphosphate group, which was transcribed *in vitro* as described previously [40].

#### Protocol RNAi screen 1 –IFNβ luciferase

In RNAi validation screen 1, we tested 187 candidate genes (**S6**and **S8 Tables**) that were predicted to play a role in the RLR signaling pathway by the computational framework. siRNAs (Dharmacon on-target plus Smartpool) were purchased internally from the Cell Screening Centre of the Utrecht University Medical Centre (CSC UMCU). Scrambled (SCR) and MDA5-targeting siRNAs were included as negative controls. Polo-like kinase 1 (PLK1)-targeting siRNAs were included as a positive control for cytotoxicity, while RIG-I-, and MAVS-targeting siRNAs were included as positive controls for RIG-I pathway activity. The RIG-I signaling pathway was activated by transfecting cells with the 5’-ppp-containing CVB3 CL RNA. Activation levels were assessed by measuring IFNβ promoter-controlled luciferase reporter activity at 6 hr post transfection (**S12 Fig**).

Screen 1 was performed in four technical replicates. Briefly, 0.5 pmole siRNAs (in 5 μl) was spotted per well. On the day of transfection 0.3 μl Lipofectamine RNAiMAX was diluted in 15 μl Opti-MEM and added to each well. Plates were rocked gently to mix the components and incubated at room temperature (RT) for 15 min. Then, 7,000 HeLa-IFNβ-Fluc cells (in 80 μl) were added to each well and plates were returned to a 37°C incubator. At 2 days post siRNA transfection, growth medium was discarded, replaced by 100 μl fresh medium and cells were then transfected with the RIG-I ligand. Briefly, 200 ng ligand and 0.8 μl Lipofectamine 2000 were separately diluted in 25 μl Opti-MEM, and incubated at RT for 5 min. These components were then mixed, incubated at RT for 20 min, and added to each well. At 6 hr post transfection, one replicate of each plate was fixed in 4% PFA and stained with DAPI. This replicate was later scanned at the CSC UMCU, and DAPI-positive nuclei were counted per well as an indication of cell viability upon siRNA transfections. The other three replicates were lysed in 30 μl 1x Passive Lysis Buffer (Promega) and allowed to freeze at -20°C. To measure luciferase activity, cell lysates were mixed by pipetting, and 15 μl from each well was transferred to a measurement plate, which was read using an automated plate reader using the following parameters: inject 40 μl firefly luciferase substrate (Promega), mix for 1 second, 1 second delay, measure for 10 seconds.

#### Protocol RNAi screen 2 –IFNβ luciferase

In RNAi validation screen 2, we tested the 57 top hits with the largest effects in screen 1 (stringent Z-score of <-2 or >2; **S8 Table**) using a different set of siRNAs, separately assessing the 42 down-hits (siRNA knockdown of which resulted in down-regulation of RIG-I-mediated IFNβ induction) and 15 up-hits (siRNA knockdown of which resulted in up-regulation of RIG-I-mediated IFNβ induction). For 48 of the 57 genes tested, siRNAs (1 pool per gene) were purchased from SIGMA (esiRNAs human library) and used at 1 pmole per well during transfection. For the remaining 9 genes, for which esiRNA products were not available, Silencer Select siRNAs were purchased from Ambion, and three oligos per gene were pooled at 1:1:1 ratio and transfected at 0.5 pmole per well.

Screen 2 was performed in six technical replicates. The protocol was in principle the same as for RNAi screen 1, except that the MTT assay using Thiazolyl Blue Tetrazolium Bromide (SIGMA) (three replicates) was used to assess cell viability instead of DAPI staining. For the MTT assay, 60 μl 80 μg/ml MTT in medium was added to each well 1 hr prior to cell harvesting. The plates were incubated to 37°C for 1 hr. MTT-containing medium was removed, and reactions were quenched by adding 150 μl DMSO per well. The resulting mixture was measured at 570 nm using a plate reader.

#### Statistical analysis of RNAi screens 1 and 2

Raw Fluc intensities (S13A and S13E Fig) displayed limited variation between plates and were normalized using a negative control-based robust Z-score [80,81], which expresses each well as the number of median absolute deviations (MAD) its intensity deviates from the median of the negative controls (non-transfected, scrambled and MDA5 siRNA wells) on the plate:

$$Robust\;Z-score(x)=\frac{x-median(negative\;controls)}{MAD(negative\;controls)}$$

Replicate plates (n = 3) were then summarized by taking the median of the robust Z-scores of the well across the three plates (S13B, S13C, S13F and S13G Fig). We observed a clear difference in IFNβ induction levels between the positive (RIG-I and MAVS) and negative controls (mock treatment, scrambled and MDA5; **Figs 2B** and **S13**). Furthermore, significant correlation exists between screens 1 and 2 (correlation between Z-scores of all 57 genes tested in both screens, including the controls: Pearson r = 0.61, *P* = 8.6 × 10^−15^).

To reduce the potential for false-positive results, toxicity of the siRNA treatment was assessed by measuring nuclei counts (DAPI staining) in screen 1 (n = 1) and cellular activity (MTT essay) in screen 2 (n = 3). Both readouts were normalized per plate by calculating the percentage of the median of the negative controls (non-transfected and scrambled wells) and clearly separated negative from positive (PLK1) toxicity controls. Only a few siRNAs reduced cell numbers by over 50% in screen 1 (**S13D Fig**). However, knockdown of none of the 57 genes tested in screen 2 reduced cellular activity by more than 50%; only COPA showed slight toxicity (MTT level compared to negative controls is 53%; **S13H Fig** and **S8 Table**). Thus, the observed effects of the siRNA knockdowns on IFNβ induction are largely independent of siRNA-induced reductions in cell numbers or cellular activity.

#### Protocol RNAi screen 3 –IFNβ mRNA

We assessed the 19 top hits (**Fig 2C** and **S8 Table**) with the consistent largest effects in both RNAi screen 1 and 2 (5’-pppRNA-induced IFNβ induction in HeLa-IFNβ-Fluc reporter cells, stringent Z-score <-2), again for an effect on *IFNβ* (*IFNB1*) mRNA expression in an independent set of experiments. For 16 of these 19 genes, siRNAs (1 pool per gene) were purchased from SIGMA (esiRNAs human library). For the other 3 genes, for which esiRNA products were not available, Silencer Select siRNAs were purchased from Ambion, and three oligos per gene were pooled at 1:1:1 ratio. This RNAi screen 3 was performed in 24-well clusters, and performed in triplicate. Briefly, 5 pmole siRNAs were diluted in 50 μl Opti-MEM and incubated 5 min at RT. Next, 1 μl Lipofectamine RNAiMAX was added and incubated another 20 min at RT. Then, 25,000 HeLa-R19 cells (in 500 μl) were added to each well and plates were returned to a 37°C incubator. At 3 days post siRNA transfection, cells were transfected with the RIG-I ligand. Briefly, 200 ng ligand and 1 μl Lipofectamine 2000 were separately diluted in 50 μl Opti-MEM, and incubated at RT for 5 min. These components were then mixed, incubated at RT for 20 min, and added to each well. At 6 hr post transfection, total cellular RNA was isolated using the NucleoSpin RNA isolation kit (Macherey-Nagel) according to manufacturer’s instructions. Isolated RNA was used for reverse transcription using the TaqMan reverse transcription reagents kit (Applied Biosystems) with random hexamers primers (Invitrogen) according to manufacturer’s instructions. Quantitative analysis of *IFNβ* mRNA levels was performed using the LightCycler 480 (Roche) as described before [82].

### Software and tools

Plots, statistics and other calculations were done using custom Perl and SQL scripts, and the R statistical package [83] with additional packages gplots [84], ROCR [85] and RNAither [86]. One-way ANOVA with Dunnett's post hoc test was performed using GraphPad Prism (GraphPad Software).

## Supporting Information

S1 Fig Overview of the 49 RLR pathway components used as positive gold standard in our study (‘RLR genes’). We focused on components that make up the intracellular core of the pathway, hence excluding the interferons and proinflammatory cytokines that are induced. The depicted network is based on the KEGG map of the RLR pathway [69]. Only key interactions are depicted. In reality, the pathway consists of a complex network of interactions [87].
(TIF)

S2 Fig Venn diagram showing the overlap between the five virus-human protein-protein interaction resources. Values represent the number of human proteins for which an interaction was reported with at least one virus. The union of the five databases (2,587 proteins) was used as a molecular signature (‘PPI with viruses’) for predicting novel RLR pathway components.
(TIF)

S3 Fig Time-course transcriptome analysis of A549 cells infected with four respiratory viruses. Cells were exposed to respiratory syncytial virus (RSV), human metapneumovirus (hMPV), parainfluenza virus (PIV), or measles virus (MV). Gene expression was measured using microarrays at 6, 12 and 24 hours after the infections. Differential expression was calculated as the log_2_ fold change comparing each infection condition to mock-infected control cells. We calculated for each gene the maximum absolute (i.e. considering both up- and down-regulation) change in expression across all time points and viruses, compared to uninfected cells. This data was used as a molecular signature (‘Differential expression upon infection’) for predicting novel RLR pathway components. (**A**) Differential expression is depicted for each gene across all infection time points. Colored lines represent the five RLR genes with the highest maximum absolute differential expression (represented by the colored dots) across all infection conditions. (**B**) Summary of the distributions of log_2_ fold changes across the infection conditions. Most genes tend to be increasingly up- or down-regulated during the course of the infection. Furthermore, RSV and hMPV generally induced much larger expression changes than PIV and MV (see **Methods**).
(TIF)

S4 Fig Analysis of the weighted co-expression calculations for the RLR pathway. (**A**) Distributions of weighted co-expression with the RLR pathway, binned into discrete intervals, for the ‘other PRR signaling pathways’ gene set (TLR, CLR, NLR, cytDNA; purple) and the set of non-RLR genes (red). Although the genome-wide *RLR co-expression scores* (x-axis in panels A-C) were calculated based on the set of known RLR genes, to avoid circularity we calculated the *likelihood ratio scores* (**Tables 1**and **S1**) of this feature (‘Co-expression with RLR pathway’) using the independent set of TLR, CLR, NLR, cytDNA genes (see **Methods**). This panel A is the same plot as the co-expression panel in **Fig 1A**. (**B**) Kernel density estimates and (**C**) boxplots of RLR co-expression scores for the various gene sets. Density estimates were calculated using a Gaussian kernel with a smoothing bandwidth given by Silverman's rule of thumb, and were normalized to 1. *P* values were calculated using the Mann-Whitney *U* test. (**D**) Recall performance (also known as sensitivity) of the weighted co-expression method for retrieving a fraction of the 49 known RLR genes (y-axis) given an inclusion cut-off rank (x-axis), across a 49x leave-one-out cross-validation (green) (see **Methods**). The recall performance of the method for other sets of genes across the cross-validation ranks is also shown to demonstrate the ability of our method to retrieve RLR genes specifically compared to other PRR pathway genes (purple), or other innate immunity genes (blue).
(TIF)

S5 Fig Distributions of the integrated RLR score for the positive (RLR genes) and negative (non-RLR genes) training sets. Integration of the 10 molecular signature data sets into the Bayesian RLR score enriches for RLR genes and depletes non-RLR genes compared to the individual data sets (see also **Figs 1A** and **S9**).
(TIF)

S6 Fig KEGG [69] pathway enrichment analysis of the top 354 RLR predictions excluding known RLR genes. Purple bars indicate PRR signaling pathways other than the RLR pathway (TLR, CLR, NLR, cytDNA), blue bars indicate additional immunity-related pathways. Enrichment was determined using the functional annotation tool of the DAVID suite version 6.7 [88] with default settings and a false discovery rate (q-value) of 0.01. Background: all human genes. See also **S7 Table**.
(TIF)

S7 Fig ClueGO [89] enrichment analysis of REACTOME pathways [90] in the top 354 RLR predictions. Nodes represent significantly enriched REACTOME terms (Bonferroni step-down corrected *P* < 0.01, background: all human genes) and are grouped (as denoted by the connecting edges) based on overlapping gene lists (connectivity measure κ > 0.4). Groups of similar terms are represented by the most prominent term(s). See also **S7 Table**.
(TIF)

S8 Fig ClueGO [89] enrichment analysis of Gene Ontology Biological Process terms in the top 354 RLR predictions. Nodes represent significantly enriched terms (Bonferroni step-down corrected *P* < 0.001, background: all human genes) and are grouped (as denoted by the connecting edges) based on overlapping gene lists (connectivity measure κ > 0.7). Groups of similar terms are represented by the most prominent term. For conciseness, clusters having less than four terms are not shown. See also **S7 Table**.
(TIF)

S9 Fig Visualization of how integration of the 10 molecular signatures enriches for RLR genes and depletes non-RLR genes. Rank plots showing the top 100 genes in (on the right) six of the individual molecular signature data sets and (on the left) in the integrated RLR score. Only the six continuous (i.e. non-binary) signatures are depicted, because ordering of genes within the two classes of the binary signatures would be arbitrary. See also **Fig 1B and 1C**.
(TIF)

S10 Fig Correlations between the ten molecular signatures used for predicting novel RLR pathway components. Heatmaps depict pairwise Spearman’s rank correlation coefficients between the values in the molecular signature data sets for positive gold standard RLR genes (**A**), and negative gold standard non-RLR genes (**B**).
(TIF)

S11 Fig Rank-order plot of the estimated false discovery rate (FDR) of the RLR predictions. The FDR was adjusted to match the expected total number of genes involved in the RLR pathway (see **Methods**). The inset shows the same plot, zoomed-in on the lower-left region, and indicates occurrences of RLR (green) and non-RLR (red) genes. RLR rank 354 corresponds to an estimated FDR of ~57%.
(TIF)

S12 Fig Pilot experiments for RNAi validation screens of candidate RLR genes. (**A**) Our essay uses HeLa-IFNβ-Fluc cells stably expressing an IFNβ promoter-controlled firefly luciferase reporter. We knocked down candidate genes using different siRNAs, transfected cells with a known small 5’-ppp-containing RIG-I RNA ligand derived from coxsackievirus [40], and measured Fluc reporter expression and cell viability after 6 hours in three technical replicates. (**B-E**) Pilot experiments for RNAi screen 1 (B-C) and RNAi screen 2 (D-E). RNAi screens 1 and 2 used a different set of siRNAs. (**B,D**) IFNβ-Fluc reporter activity after treatment of HeLa-IFNβ-Fluc cells with the 5’-ppp-containing RIG-I RNA ligand and various siRNAs. Scrambled (SCR) and MDA5-targeting siRNAs were included as negative controls. Polo-like kinase 1 (PLK1)-targeting siRNAs were included as a positive control for cellular toxicity, while RIG-I-, and MAVS-targeting siRNAs were included as positive controls for RIG-I pathway activity. RNAiMax and Non-treated indicate treatment of cells without siRNA transfection (non-transfected). This setup led to specific activation of RIG-I, as RIG-I or MAVS siRNA transfection, but not MDA5 or scrambled siRNAs, resulted in loss of luciferase reporter activity. (**C,E**) As an indication of cell viability upon siRNA transfection, the number of nuclei per well (DAPI staining) was counted in screen 1 (C) or MTT activity was measured to assess cellular activity in screen 2 (E). Only the death-control PLK1 severely reduced nuclei numbers.
(TIF)

S13 Fig Analysis of RNAi screens 1 (A-D) and 2 (E-H) for validation of the candidate RLR genes. See also **Fig 2**. (**A,E**) Q-Q plots (left) of the raw luciferase intensities against the quantiles of a theoretical normal distribution (plotted by RNAither [86]). Linearity suggests that the raw data resemble a normal distribution. Boxplots (right) show the distributions of the raw luciferase intensities for the positive controls (RIG-I and MAVS siRNAs; green), negative controls (non-transfected, scrambled, and MDA5 siRNAs; red), and RLR candidates (gray). (**B,F**) Q-Q plots and boxplots of the normalized data, summarized over the replicate plates. Raw luciferase intensities were normalized using a negative control-based robust Z-score and summarized across replicate plates by taking the median Z-score (see **Methods**). Note that the gray distributions in (A-B and E-F) include the death control PLK1, which always has a luciferase signal close to zero. This causes some of the observed deviations from the normal distribution at the lower extremes, and causes the boxplots to lie a little lower than would be the case without PLK1. (**C,G**) Z-score distributions. Dotted lines indicate Z-score cutoffs of -1.25 and 1.25. Dashed lines indicate stringent Z-score cutoffs of -2 and 2. Numbers to the right of the plots indicate the number of candidate RLR genes scoring within the indicated Z-score range. Knockdown of 94 genes of the 187 tested candidates (50%) affected RIG-I-mediated IFNβ induction at Z-score <-1.25 or >1.25 in RNAi screen 1, of which 59 decreased and 35 increased IFNβ induction. The 57 top hits with stringent Z-score <-2 or >2 in screen 1 were tested again in screen 2 using a different set of siRNAs (**Fig 2A**). (**D,H**) Z-score (left y-axis) versus cell count (nuclei staining, right y-axis in (D)) or cellular activity (measured by MTT essay, right y-axis in (H)) distributions. Cell counts and MTT essay are presented as the percentage of the median of the negative controls (non-transfected and scrambled wells). No correlation exists between the effects of gene knockdown on the luciferase activity Z-score and cellular toxicity. All data points close to 0% cell counts or MTT are from the positive toxicity control PLK1.
(TIF)

S14 Fig No molecular signature solely explains the predictions of the experimentally validated hits. Distributions of the 187 candidate RLR genes selected for experimental validation, across the 10 molecular signature data sets we identified as predictive of the RLR system (see also **Fig 1A**). RLR candidates were grouped based on the results from RNAi screen 1: no hit (gray), all hits from RNAi screen 1 (94 hits with Z-score <-1.25 or >1.25, dark purple), and top hits from RNAi screen 1 (57 hits with Z-score <-2 or >2, purple) (see also **Fig 2**). Fractions of genes in the same group add up to one. ‘NA’ bins represent genes for which there was no data in the respective molecular signature (note that these bins did not receive a score in the Bayesian integration, see **Methods**).
(TIF)

S1 Table Likelihood scores for the 10 molecular signatures of RLR genes. Note that, to avoid circularity, the predictive ability of the co-expression, protein domain and RLR pathway PPI data sets was assessed using the set of TLR, CLR, NLR, cytDNA genes instead of the RLR genes (see **Methods**).
(XLSX)

S2 Table List of the 128 viral miRNAs for which we obtained predicted target sites in human mRNAs. (XLSX)

S3 Table Meta analysis of antiviral host factors from published RNAi screens. (XLSX)

S4 Table Enrichment analysis of protein domains occurring in RLR pathway components. (XLSX)

S5 Table Enrichment analysis of conserved IRF, AP–1, NFκB, and STAT TF binding motifs in the promoters of RLR pathway genes. (XLSX)

S6 Table Genome-wide prioritization of RLR pathway components based on the integrated RLR score. Also available at http://rlr.cmbi.umcn.nl/.
(XLSX)

S7 Table Function enrichment analysis of the top 354 RLR predictions excluding known RLR genes. Function enrichment (gene ontology, pathways, disease) was determined using the functional annotation tool of the DAVID suite version 6.7 [88] with default settings and a false discovery rate (q-value) of 0.01. In cases where multiple function terms form a cluster at medium stringency according to the ‘Functional Annotation Clustering’ view, only the term with the lowest q-value is shown for conciseness. Background: all human genes.
(XLSX)

S8 Table Detailed results of the RNAi validation screens. (XLSX)

S9 Table Impact of the prior on the RLR score and false discovery rate. (XLSX)
