# Supplementary figures and images for: Integrative Genomics-Based Discovery of Novel Regulators of the Innate Antiviral Response
Source: PLoS Comput Biol. 2015 Oct 20;11(10):e1004553. doi: 10.1371/journal.pcbi.1004553 (PMC4618338; doi:10.1371/journal.pcbi.1004553)

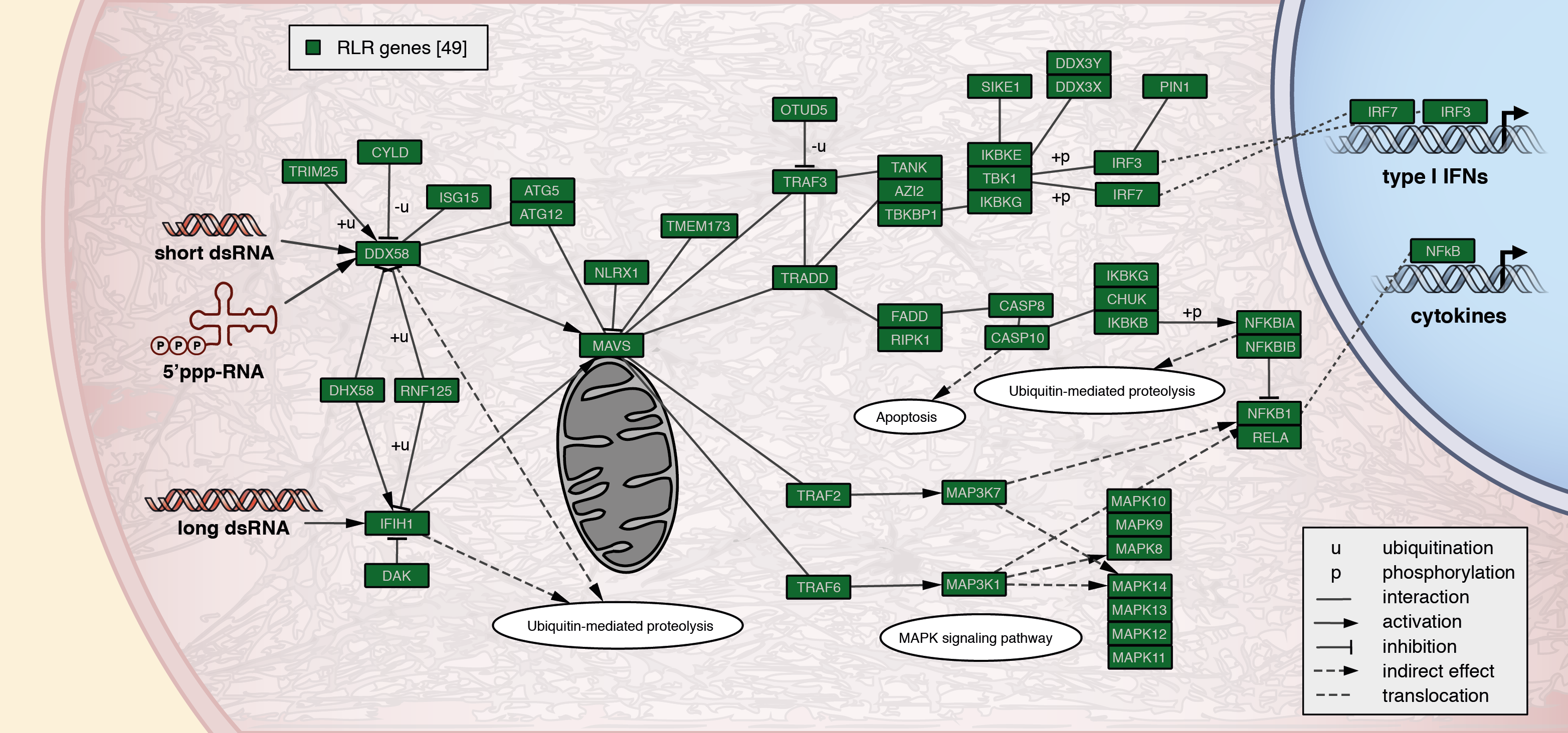

Supplement: S1 Fig — We focused on components that make up the intracellular core of the pathway, hence excluding the interferons and proinflammatory cytokines that are induced. The depicted network is based on the KEGG map of the RLR pathway [69]. Only key interactions are depicted. In reality, the pathway consists of a complex network of interactions [87]. (TIF) [file pcbi.1004553.s001.tif]

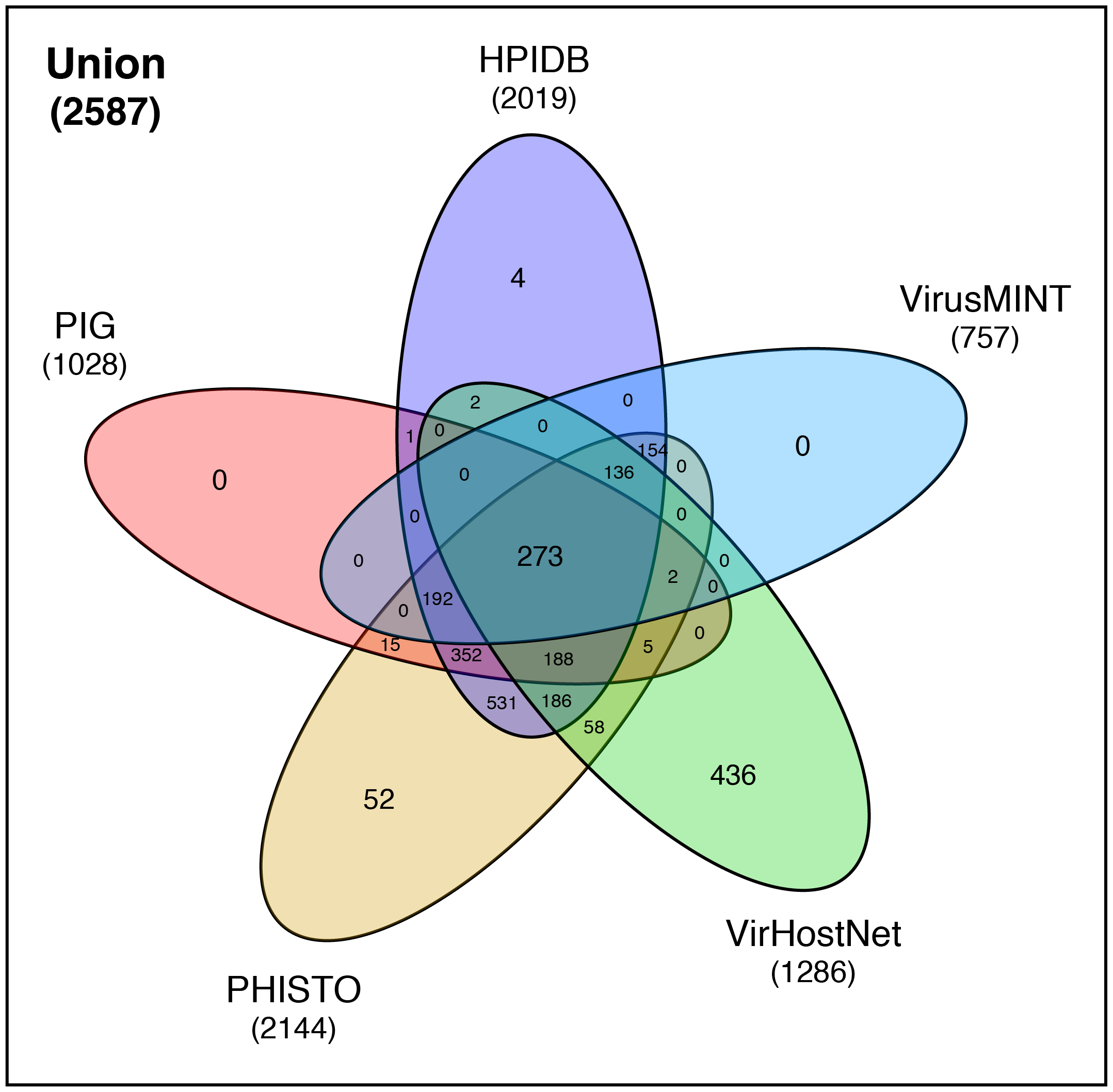

Supplement: S2 Fig — Values represent the number of human proteins for which an interaction was reported with at least one virus. The union of the five databases (2,587 proteins) was used as a molecular signature (‘PPI with viruses’) for predicting novel RLR pathway components. (TIF) [file pcbi.1004553.s002.tif]

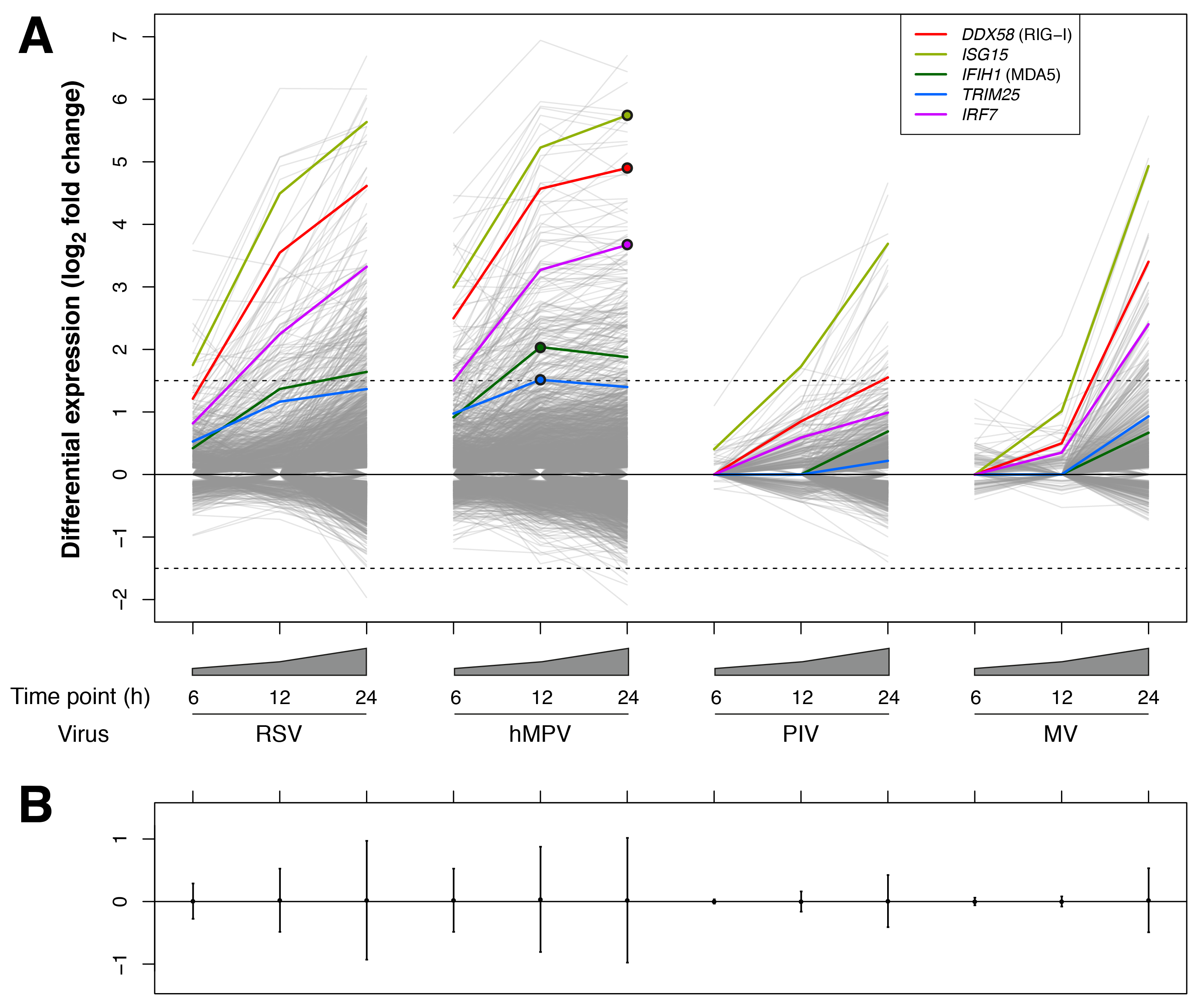

Supplement: S3 Fig — Cells were exposed to respiratory syncytial virus (RSV), human metapneumovirus (hMPV), parainfluenza virus (PIV), or measles virus (MV). Gene expression was measured using microarrays at 6, 12 and 24 hours after the infections. Differential expression was calculated as the log2 fold change comparing each infection condition to mock-infected control cells. We calculated for each gene the maximum absolute (i.e. considering both up- and down-regulation) change in expression across all time points and viruses, compared to uninfected cells. This data was used as a molecular signature (‘Differential expression upon infection’) for predicting novel RLR pathway components. (A) Differential expression is depicted for each gene across all infection time points. Colored lines represent the five RLR genes with the highest maximum absolute differential expression (represented by the colored dots) across all infection conditions. (B) Summary of the distributions of log2 fold changes across the infection conditions. Most genes tend to be increasingly up- or down-regulated during the course of the infection. Furthermore, RSV and hMPV generally induced much larger expression changes than PIV and MV (see Methods). (TIF) [file pcbi.1004553.s003.tif]

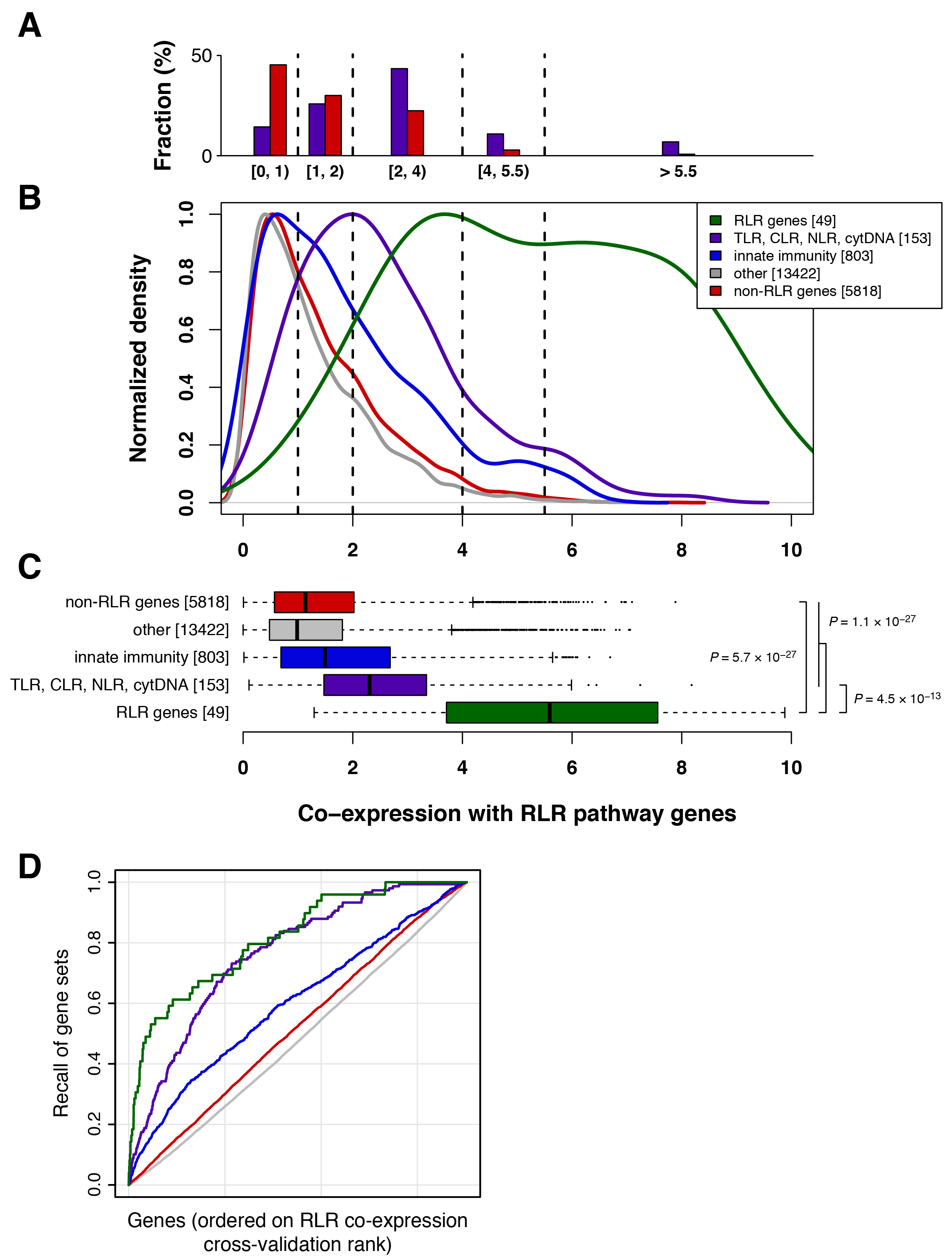

Supplement: S4 Fig — (A) Distributions of weighted co-expression with the RLR pathway, binned into discrete intervals, for the ‘other PRR signaling pathways’ gene set (TLR, CLR, NLR, cytDNA; purple) and the set of non-RLR genes (red). Although the genome-wide RLR co-expression scores (x-axis in panels A-C) were calculated based on the set of known RLR genes, to avoid circularity we calculated the likelihood ratio scores (Tables 1and S1) of this feature (‘Co-expression with RLR pathway’) using the independent set of TLR, CLR, NLR, cytDNA genes (see Methods). This panel A is the same plot as the co-expression panel in Fig 1A. (B) Kernel density estimates and (C) boxplots of RLR co-expression scores for the various gene sets. Density estimates were calculated using a Gaussian kernel with a smoothing bandwidth given by Silverman's rule of thumb, and were normalized to 1. P values were calculated using the Mann-Whitney U test. (D) Recall performance (also known as sensitivity) of the weighted co-expression method for retrieving a fraction of the 49 known RLR genes (y-axis) given an inclusion cut-off rank (x-axis), across a 49x leave-one-out cross-validation (green) (see Methods). The recall performance of the method for other sets of genes across the cross-validation ranks is also shown to demonstrate the ability of our method to retrieve RLR genes specifically compared to other PRR pathway genes (purple), or other innate immunity genes (blue). (TIF) [file pcbi.1004553.s004.tif]

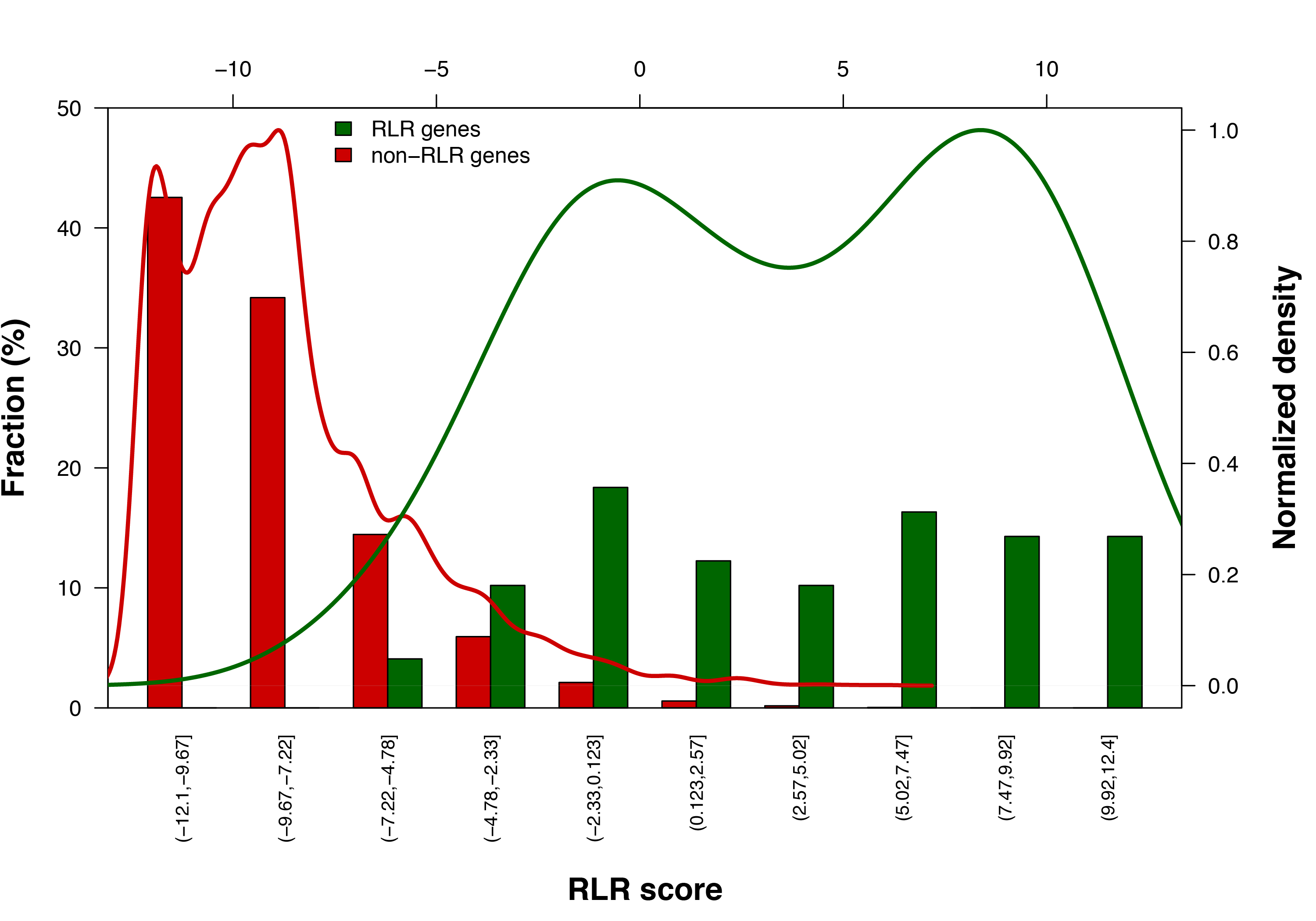

Supplement: S5 Fig — Integration of the 10 molecular signature data sets into the Bayesian RLR score enriches for RLR genes and depletes non-RLR genes compared to the individual data sets (see also Figs 1A and S9). (TIF) [file pcbi.1004553.s005.tif]

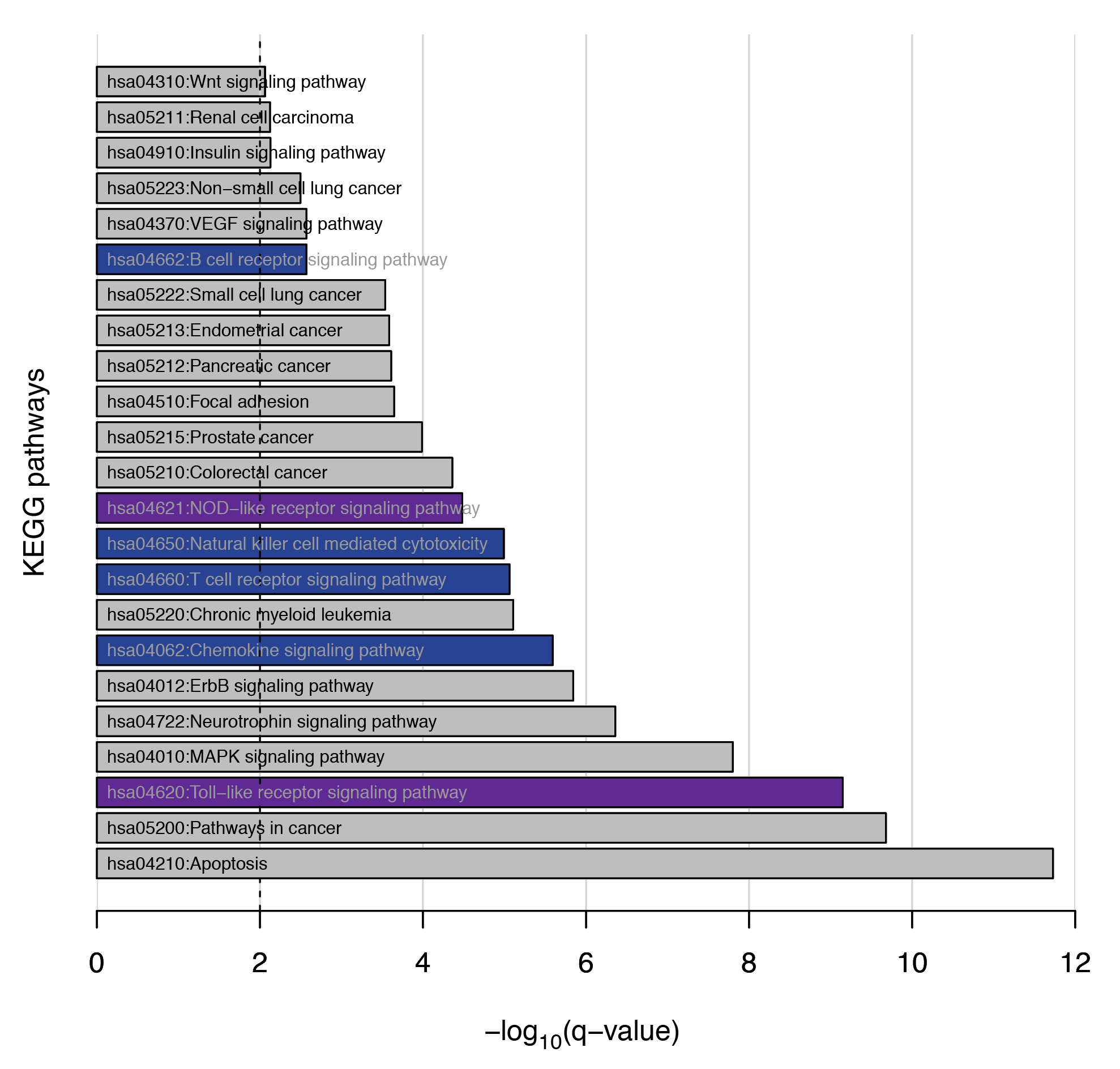

Supplement: S6 Fig — Purple bars indicate PRR signaling pathways other than the RLR pathway (TLR, CLR, NLR, cytDNA), blue bars indicate additional immunity-related pathways. Enrichment was determined using the functional annotation tool of the DAVID suite version 6.7 [88] with default settings and a false discovery rate (q-value) of 0.01. Background: all human genes. See also S7 Table. (TIF) [file pcbi.1004553.s006.tif]

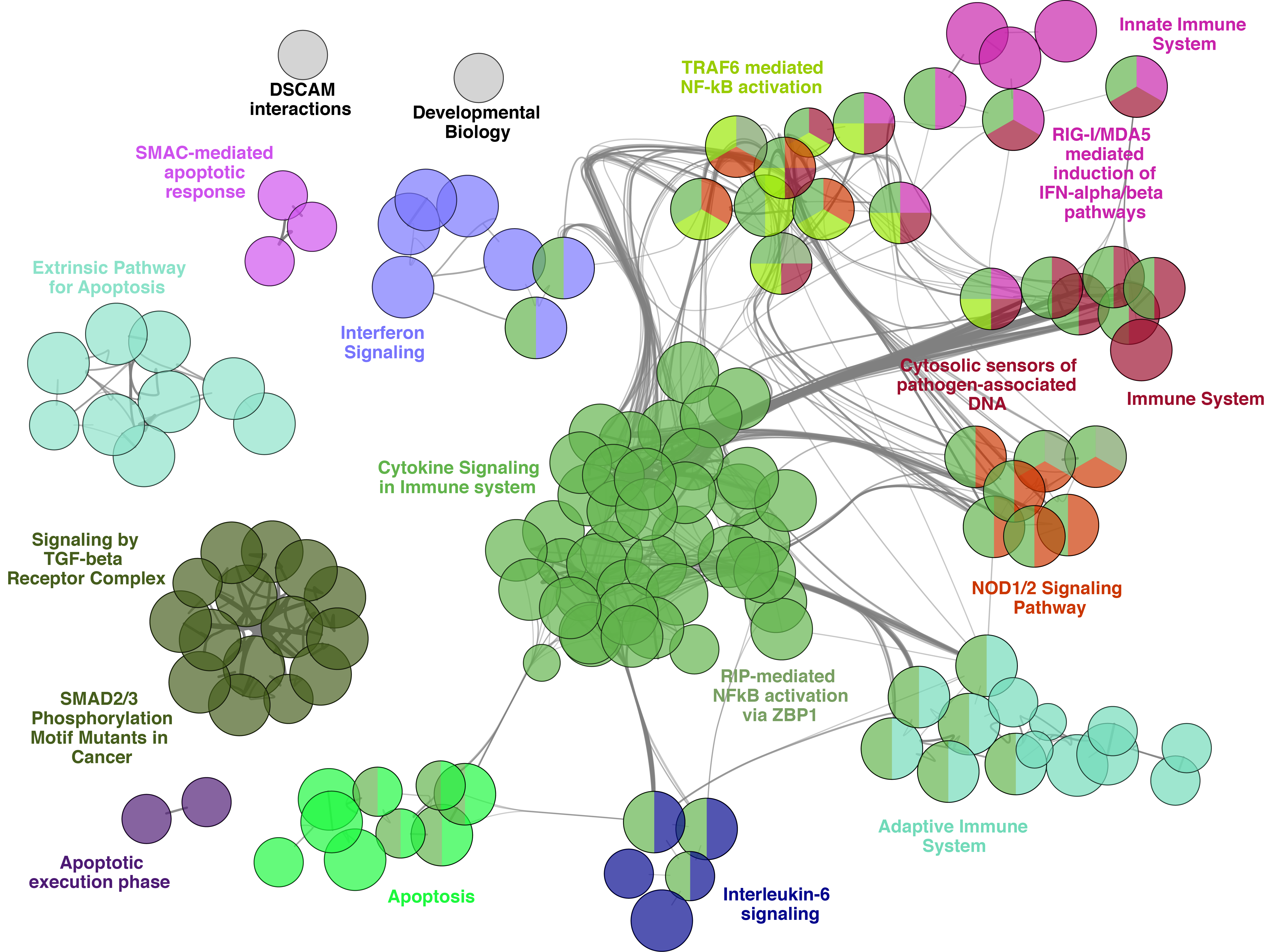

Supplement: S7 Fig — Nodes represent significantly enriched REACTOME terms (Bonferroni step-down corrected P < 0.01, background: all human genes) and are grouped (as denoted by the connecting edges) based on overlapping gene lists (connectivity measure κ > 0.4). Groups of similar terms are represented by the most prominent term(s). See also S7 Table. (TIF) [file pcbi.1004553.s007.tif]

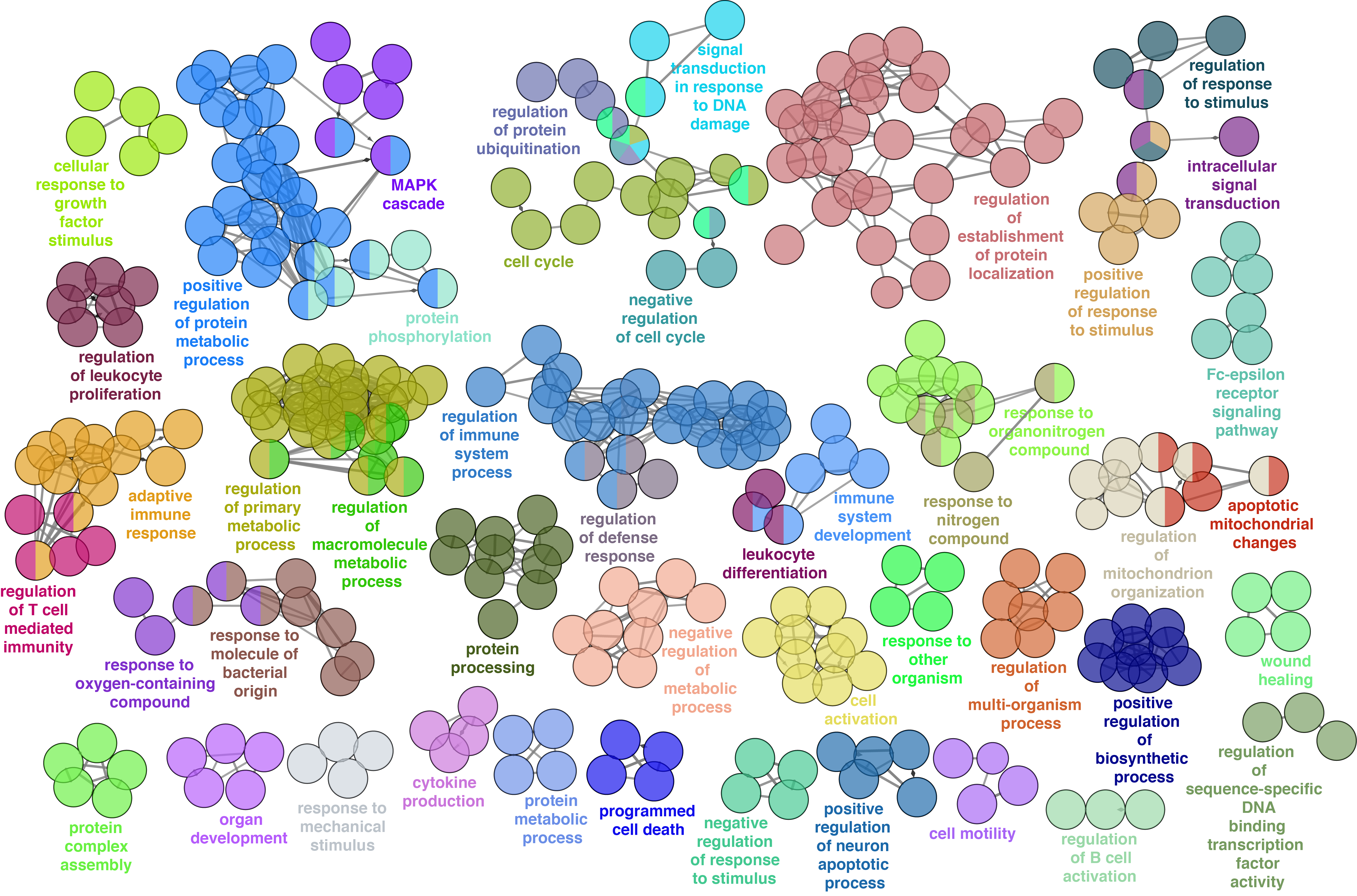

Supplement: S8 Fig — Nodes represent significantly enriched terms (Bonferroni step-down corrected P < 0.001, background: all human genes) and are grouped (as denoted by the connecting edges) based on overlapping gene lists (connectivity measure κ > 0.7). Groups of similar terms are represented by the most prominent term. For conciseness, clusters having less than four terms are not shown. See also S7 Table. (TIF) [file pcbi.1004553.s008.tif]

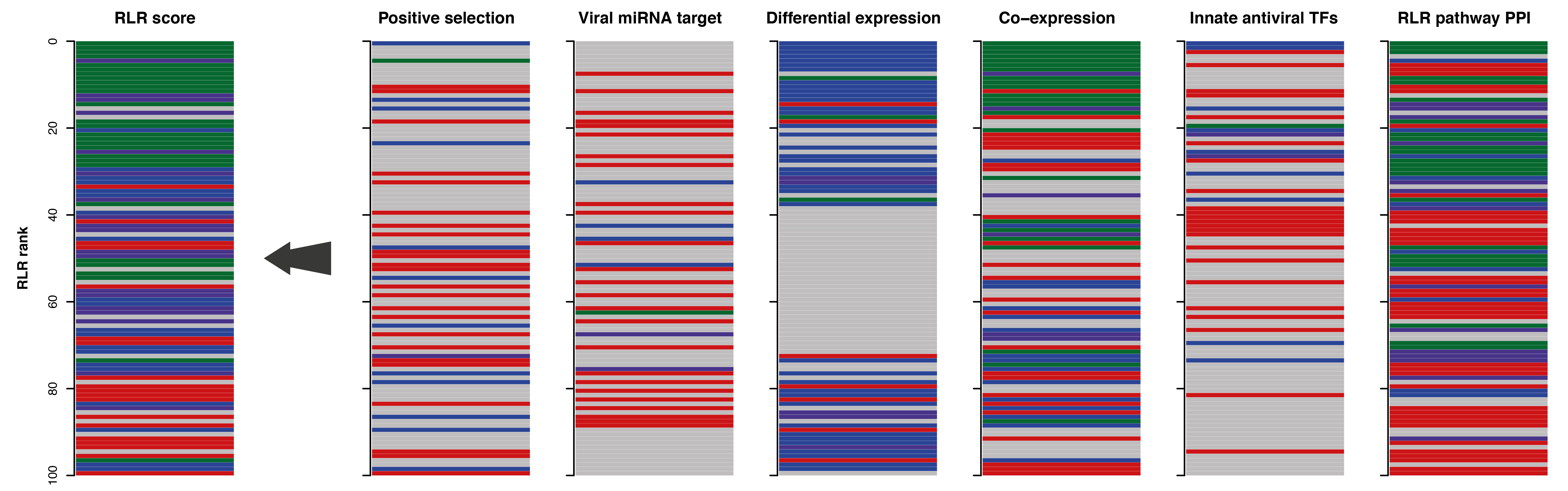

Supplement: S9 Fig — Rank plots showing the top 100 genes in (on the right) six of the individual molecular signature data sets and (on the left) in the integrated RLR score. Only the six continuous (i.e. non-binary) signatures are depicted, because ordering of genes within the two classes of the binary signatures would be arbitrary. See also Fig 1B and 1C. (TIF) [file pcbi.1004553.s009.tif]

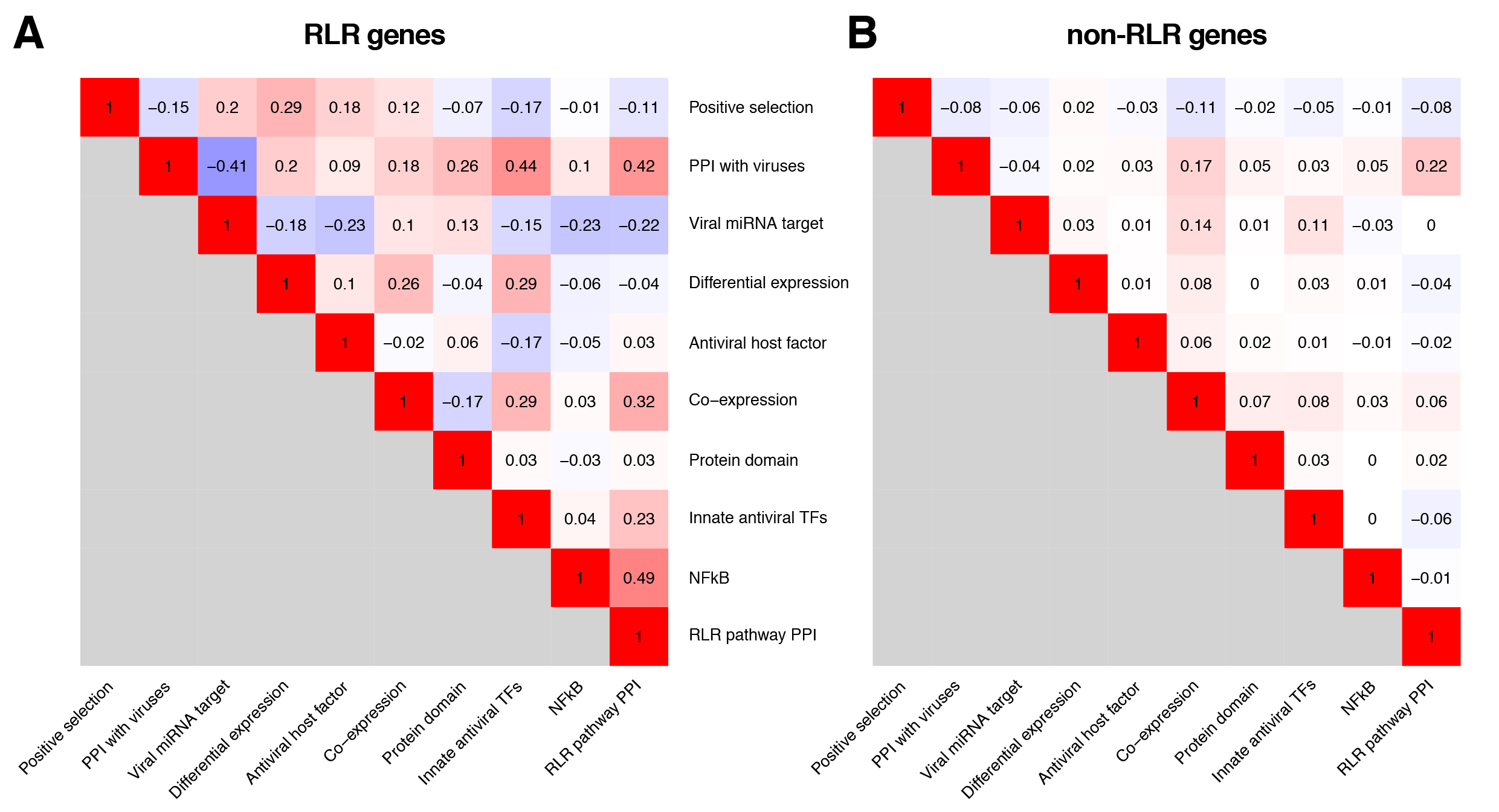

Supplement: S10 Fig — Heatmaps depict pairwise Spearman’s rank correlation coefficients between the values in the molecular signature data sets for positive gold standard RLR genes (A), and negative gold standard non-RLR genes (B). (TIF) [file pcbi.1004553.s010.tif]

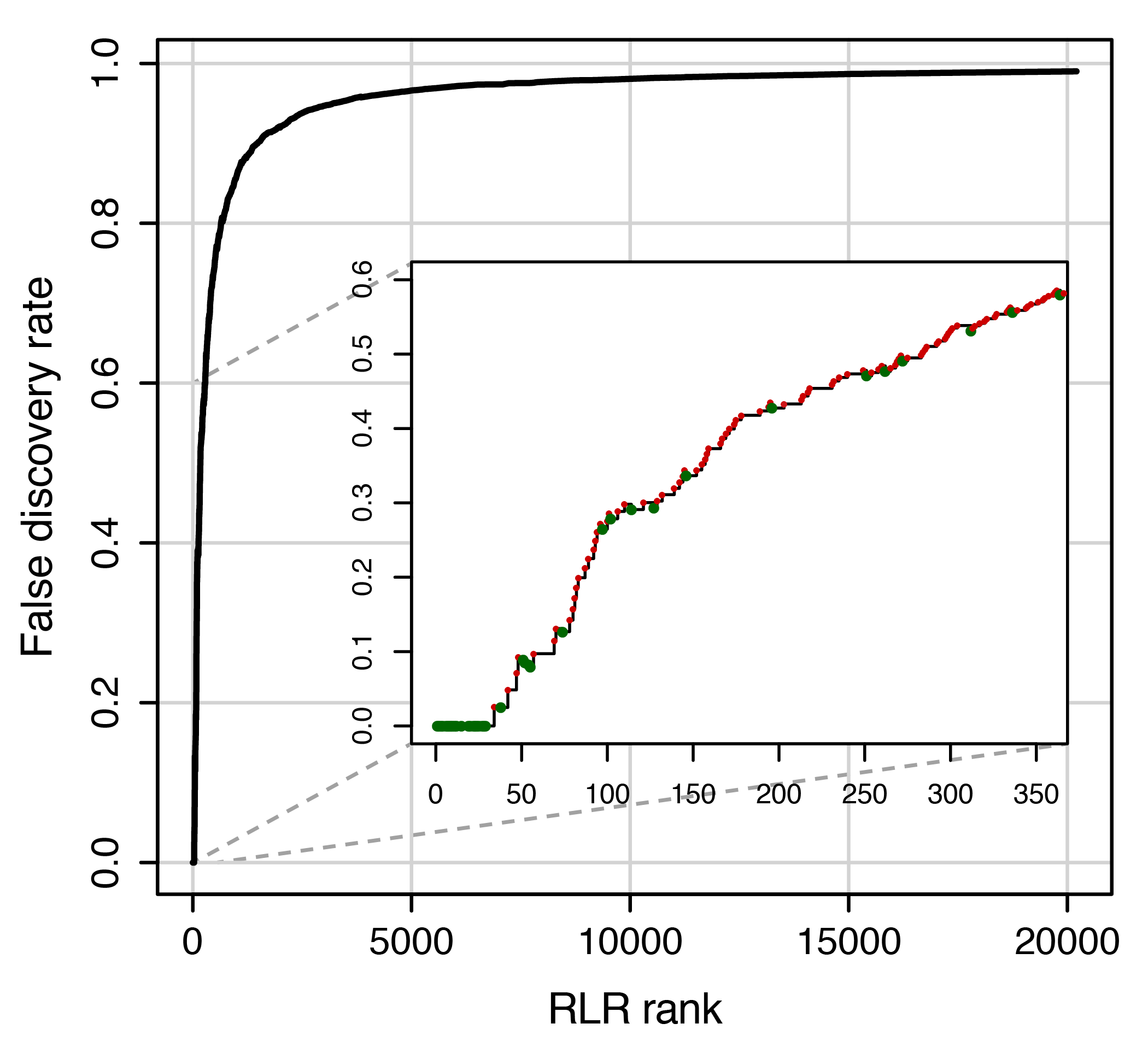

Supplement: S11 Fig — The FDR was adjusted to match the expected total number of genes involved in the RLR pathway (see Methods). The inset shows the same plot, zoomed-in on the lower-left region, and indicates occurrences of RLR (green) and non-RLR (red) genes. RLR rank 354 corresponds to an estimated FDR of ~57%. (TIF) [file pcbi.1004553.s011.tif]

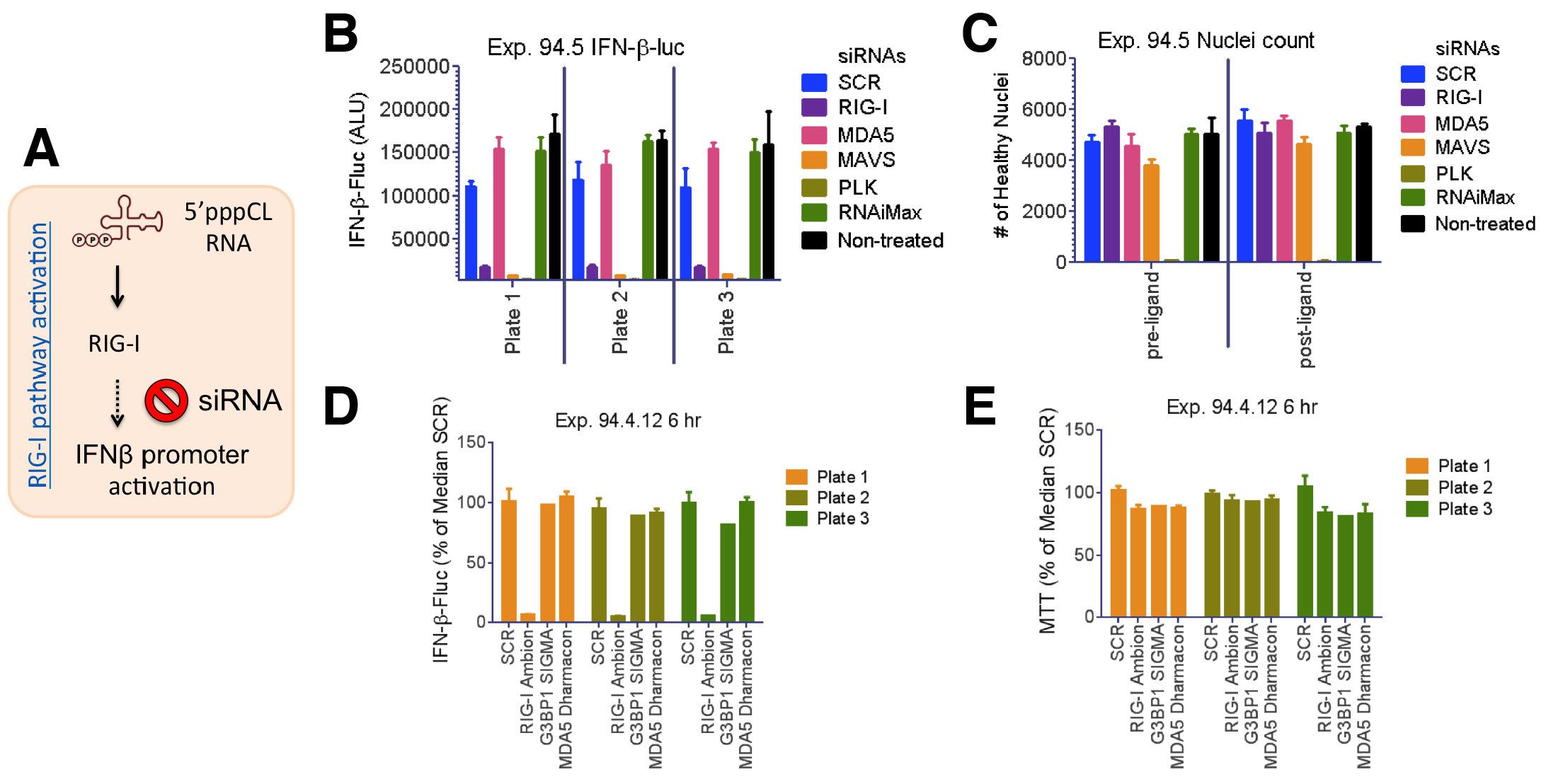

Supplement: S12 Fig — (A) Our essay uses HeLa-IFNβ-Fluc cells stably expressing an IFNβ promoter-controlled firefly luciferase reporter. We knocked down candidate genes using different siRNAs, transfected cells with a known small 5’-ppp-containing RIG-I RNA ligand derived from coxsackievirus [40], and measured Fluc reporter expression and cell viability after 6 hours in three technical replicates. (B-E) Pilot experiments for RNAi screen 1 (B-C) and RNAi screen 2 (D-E). RNAi screens 1 and 2 used a different set of siRNAs. (B,D) IFNβ-Fluc reporter activity after treatment of HeLa-IFNβ-Fluc cells with the 5’-ppp-containing RIG-I RNA ligand and various siRNAs. Scrambled (SCR) and MDA5-targeting siRNAs were included as negative controls. Polo-like kinase 1 (PLK1)-targeting siRNAs were included as a positive control for cellular toxicity, while RIG-I-, and MAVS-targeting siRNAs were included as positive controls for RIG-I pathway activity. RNAiMax and Non-treated indicate treatment of cells without siRNA transfection (non-transfected). This setup led to specific activation of RIG-I, as RIG-I or MAVS siRNA transfection, but not MDA5 or scrambled siRNAs, resulted in loss of luciferase reporter activity. (C,E) As an indication of cell viability upon siRNA transfection, the number of nuclei per well (DAPI staining) was counted in screen 1 (C) or MTT activity was measured to assess cellular activity in screen 2 (E). Only the death-control PLK1 severely reduced nuclei numbers. (TIF) [file pcbi.1004553.s012.tif]

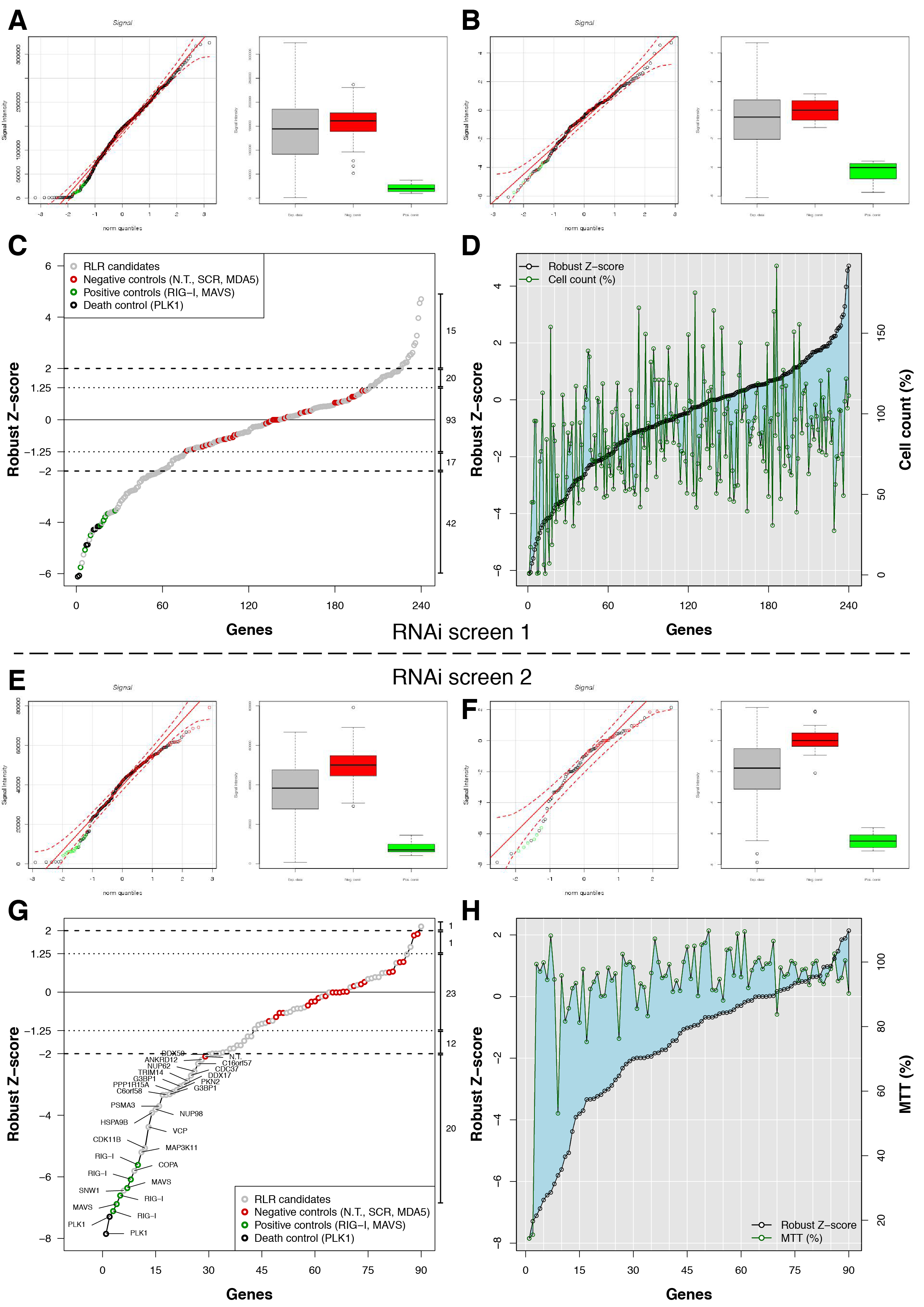

Supplement: S13 Fig — See also Fig 2. (A,E) Q-Q plots (left) of the raw luciferase intensities against the quantiles of a theoretical normal distribution (plotted by RNAither [86]). Linearity suggests that the raw data resemble a normal distribution. Boxplots (right) show the distributions of the raw luciferase intensities for the positive controls (RIG-I and MAVS siRNAs; green), negative controls (non-transfected, scrambled, and MDA5 siRNAs; red), and RLR candidates (gray). (B,F) Q-Q plots and boxplots of the normalized data, summarized over the replicate plates. Raw luciferase intensities were normalized using a negative control-based robust Z-score and summarized across replicate plates by taking the median Z-score (see Methods). Note that the gray distributions in (A-B and E-F) include the death control PLK1, which always has a luciferase signal close to zero. This causes some of the observed deviations from the normal distribution at the lower extremes, and causes the boxplots to lie a little lower than would be the case without PLK1. (C,G) Z-score distributions. Dotted lines indicate Z-score cutoffs of -1.25 and 1.25. Dashed lines indicate stringent Z-score cutoffs of -2 and 2. Numbers to the right of the plots indicate the number of candidate RLR genes scoring within the indicated Z-score range. Knockdown of 94 genes of the 187 tested candidates (50%) affected RIG-I-mediated IFNβ induction at Z-score <-1.25 or >1.25 in RNAi screen 1, of which 59 decreased and 35 increased IFNβ induction. The 57 top hits with stringent Z-score <-2 or >2 in screen 1 were tested again in screen 2 using a different set of siRNAs (Fig 2A). (D,H) Z-score (left y-axis) versus cell count (nuclei staining, right y-axis in (D)) or cellular activity (measured by MTT essay, right y-axis in (H)) distributions. Cell counts and MTT essay are presented as the percentage of the median of the negative controls (non-transfected and scrambled wells). No correlation exists between the effects of gene knockdown on the [file pcbi.1004553.s013.tif]

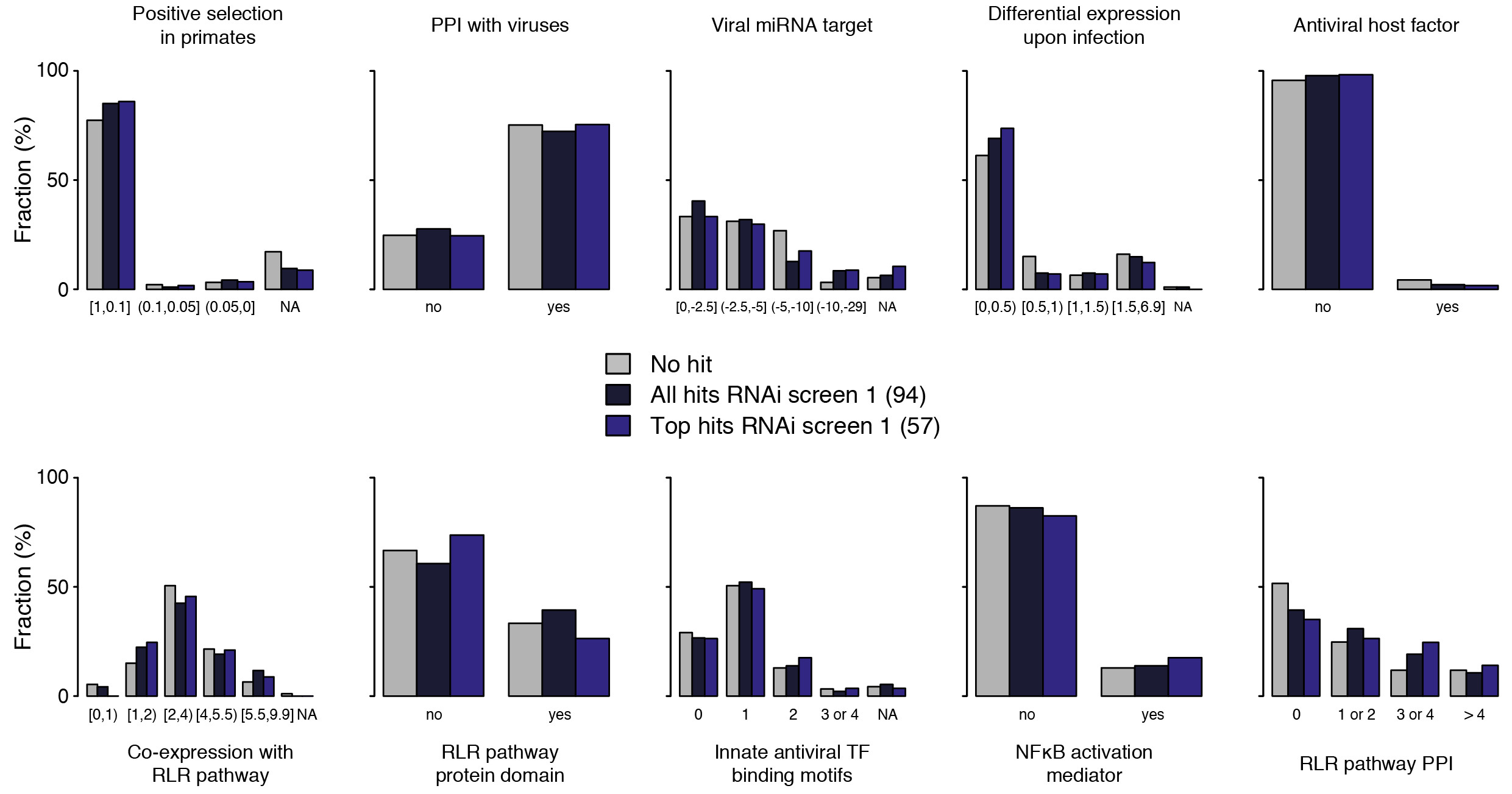

Supplement: S14 Fig — Distributions of the 187 candidate RLR genes selected for experimental validation, across the 10 molecular signature data sets we identified as predictive of the RLR system (see also Fig 1A). RLR candidates were grouped based on the results from RNAi screen 1: no hit (gray), all hits from RNAi screen 1 (94 hits with Z-score <-1.25 or >1.25, dark purple), and top hits from RNAi screen 1 (57 hits with Z-score <-2 or >2, purple) (see also Fig 2). Fractions of genes in the same group add up to one. ‘NA’ bins represent genes for which there was no data in the respective molecular signature (note that these bins did not receive a score in the Bayesian integration, see Methods). (TIF) [file pcbi.1004553.s014.tif]
